# Association of Hemoglobin A_1c_ Levels With Use of Sulfonylureas, Dipeptidyl Peptidase 4 Inhibitors, and Thiazolidinediones in Patients With Type 2 Diabetes Treated With Metformin: Analysis From the Observational Health Data Sciences and Informatics Initiative

**DOI:** 10.1001/jamanetworkopen.2018.1755

**Published:** 2018-08-24

**Authors:** Rohit Vashisht, Kenneth Jung, Alejandro Schuler, Juan M. Banda, Rae Woong Park, Sanghyung Jin, Li Li, Joel T. Dudley, Kipp W. Johnson, Mark M. Shervey, Hua Xu, Yonghui Wu, Karthik Natrajan, George Hripcsak, Peng Jin, Mui Van Zandt, Anthony Reckard, Christian G. Reich, James Weaver, Martijn J. Schuemie, Patrick B. Ryan, Alison Callahan, Nigam H. Shah

**Affiliations:** 1 Observational Health Data Sciences and Informatics, New York, New York; 2 Center for Biomedical Informatics Research, Stanford University School of Medicine, Stanford, California; 3 Department of Biomedical Sciences, Ajou University Graduate School of Medicine, Suwon, Gyeonggi-do, Republic of Korea; 4 Department of Biomedical Informatics, Ajou University School of Medicine, Suwon, Gyeonggi-do, Republic of Korea; 5 The Institute of Next Generation of Healthcare, Icahn School of Medicine at Mount Sinai, New York, New York; 6 School of Biomedical Informatics, The University of Texas Health Science Center at Houston, Houston; 7 Department of Health Outcome and Policy, College of Medicine, University of Florida, Gainesville; 8 New York–Presbyterian Hospital, New York; 9 Department of Biomedical Informatics, Columbia University, New York, New York; 10 IQVIA, Durham, North Carolina; 11 Janssen Research and Development, Raritan, New Jersey

## Abstract

**Importance:**

Consensus around an efficient second-line treatment option for type 2 diabetes (T2D) remains ambiguous. The availability of electronic medical records and insurance claims data, which capture routine medical practice, accessed via the Observational Health Data Sciences and Informatics network presents an opportunity to generate evidence for the effectiveness of second-line treatments.

**Objective:**

To identify which drug classes among sulfonylureas, dipeptidyl peptidase 4 (DPP-4) inhibitors, and thiazolidinediones are associated with reduced hemoglobin A_1c_ (HbA_1c_) levels and lower risk of myocardial infarction, kidney disorders, and eye disorders in patients with T2D treated with metformin as a first-line therapy.

**Design, Setting, and Participants:**

Three retrospective, propensity-matched, new-user cohort studies with replication across 8 sites were performed from 1975 to 2017. Medical data of 246 558 805 patients from multiple countries from the Observational Health Data Sciences and Informatics (OHDSI) initiative were included and medical data sets were transformed into a unified common data model, with analysis done using open-source analytical tools. Participants included patients with T2D receiving metformin with at least 1 prior HbA_1c_ laboratory test who were then prescribed either sulfonylureas, DPP-4 inhibitors, or thiazolidinediones. Data analysis was conducted from 2015 to 2018.

**Exposures:**

Treatment with sulfonylureas, DPP-4 inhibitors, or thiazolidinediones starting at least 90 days after the initial prescription of metformin.

**Main Outcomes and Measures:**

The primary outcome is the first observation of the reduction of HbA_1c_ level to 7% of total hemoglobin or less after prescription of a second-line drug. Secondary outcomes are myocardial infarction, kidney disorder, and eye disorder after prescription of a second-line drug.

**Results:**

A total of 246 558 805 patients (126 977 785 women [51.5%]) were analyzed. Effectiveness of sulfonylureas, DPP-4 inhibitors, and thiazolidinediones prescribed after metformin to lower HbA_1c_ level to 7% or less of total hemoglobin remained indistinguishable in patients with T2D. Patients treated with sulfonylureas compared with DPP-4 inhibitors had a small increased consensus hazard ratio of myocardial infarction (1.12; 95% CI, 1.02-1.24) and eye disorders (1.15; 95% CI, 1.11-1.19) in the meta-analysis. Hazard of observing kidney disorders after treatment with sulfonylureas, DPP-4 inhibitors, or thiazolidinediones was equally likely.

**Conclusions and Relevance:**

The examined drug classes did not differ in lowering HbA_1c_ and in hazards of kidney disorders in patients with T2D treated with metformin as a first-line therapy. Sulfonylureas had a small, higher observed hazard of myocardial infarction and eye disorders compared with DPP-4 inhibitors in the meta-analysis. The OHDSI collaborative network can be used to conduct a large international study examining the effectiveness of second-line treatment choices made in clinical management of T2D.

## Key Points

**Question:**

Can the effectiveness of second-line treatment of type 2 diabetes after initial therapy with metformin be characterized via an open collaborative research network?

**Findings:**

In this analysis of data from more than 246 million patients in multiple cohorts, treatment with dipeptidyl peptidase 4 inhibitors compared with sulfonylureas and thiazolidinediones did not differ in reducing hemoglobin A_1c_ levels or hazard of kidney disorders. In a meta-analysis, sulfonylureas compared with dipeptidyl peptidase 4 inhibitors were associated with a small increased hazard of myocardial infarction and eye disorders in patients with type 2 diabetes.

**Meaning:**

Large-scale characterization of the effectiveness of type 2 diabetes therapy across nations through an open collaborative research network aligns with the 2017 recommendation of the American Association of Clinical Endocrinologists and American College of Endocrinology in type 2 diabetes management recommending dipeptidyl peptidase 4 inhibitors over sulfonylureas in patients with diabetes for whom metformin was the first-line treatment.

## Teaser

This multinational cohort study examines the association of second-line treatment (sulfonylureas, dipeptidyl peptidase 4 inhibitors [DPP-4], or thiazolidinediones) for type 2 diabetes after initial therapy with metformin with hemoglobin A_1c_ (HbA_1c_) levels.

## Introduction

Diabetes affects 29 million people in the United States and 420 million worldwide.^1,2^ The global prevalence of diabetes will reach 642 million patients by 2040, challenging health care systems and economies.^2^ In addition, patients with diabetes often develop complications related to kidney failure, cardiovascular disorders, and blindness that reduce their quality of life and increase financial burden.^2,3,4,5^

Unless contraindicated, patients with type 2 diabetes (T2D) are prescribed metformin as first-line therapy according to existing treatment guidelines.^6,7^ However, if T2D remains uncontrolled, a second-line drug must be chosen from the multiple options available such as sulfonylureas, dipeptidyl peptidase 4 (DPP-4) inhibitors, α-glucosidase inhibitors, sodium-glucose cotransporter 2 inhibitors, glucagon-like peptide 1 receptor agonists, and thiazolidinediones.^6,7^ Given the infeasibility of conducting randomized clinical trials for every situation, and the relative availability of electronic medical records (EMRs) as well as insurance claims data, we have an opportunity to generate evidence from the record of routine clinical practice to inform this choice.^8^

The Observational Health Data Sciences and Informatics (OHDSI) initiative is an international collaborative to investigate the value of analyzing health data at scale.^9^ In the past, this group characterized treatment choices in terms of the combination of therapies and their changes over time, as well as across different locations and practice types for T2D, hypertension, and depression.^10^ In that study, metformin was the most commonly prescribed medication for diabetes; it was prescribed 75% of the time as the first medication and remained the only medication 29% of the time, thus confirming general adoption of the recommendations of the American Association of Clinical Endocrinologists and American Diabetes Association.^7,11^ However, second-line therapy varied widely, which is not surprising given the lack of consensus around second-line therapy choice.^12,13^

## Methods

### Study Population and Data Collection

**Figure 1.**
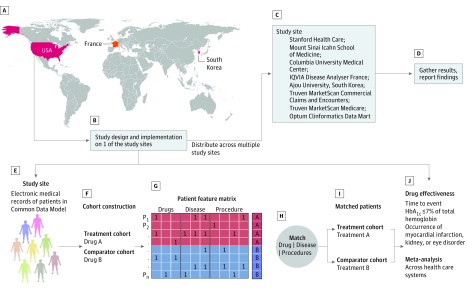
Overview of Multinational Cohort Study Design A, Countries represented in this analysis. B, The study was designed at Stanford University. C, The analysis pipeline was executed at other participating sites. D, Results from each site were synthesized into consensus estimates via a meta-analysis. E, Patient data at all study sites were transformed into the Observational Medical Outcomes Partnership Common Data Model. F-I, Construction of analysis cohorts with comprehensive patient covariate data (including drug prescriptions, disease diagnosis, demographics, and procedures), and matching based on propensity scores. G, The patients feature matrix is a representation of patient medical records. Each row in the patients feature matrix represents a patient (P_1_ to P_n_) and each column represents a drug, disease diagnosis, or procedure. A value of 1 in a cell indicates that a drug prescription, disease diagnosis, or procedure was noted in the medical record of that patient. A and B are features of interest for our study, eg, whether a patient was prescribed a dipeptidyl peptidase 4 inhibitor or a sulfonylurea. J, Effect estimation for reduction in hemoglobin A_1c_ (HbA_1c_) to 7% of total hemoglobin or less (to convert to proportion of total hemoglobin, multiply by 0.01), myocardial infarction, kidney disorders, and eye disorders.

We examined the effectiveness of second-line treatments for T2D—after first-line treatment with metformin—using data from the OHDSI collaborative research network. We performed a retrospective analysis of clinical data from more than 246 million patients across 8 data sources spanning multiple health care systems in 3 countries (Figure 1). Patient-level data from each site were transformed into a common data schema that enabled identical study execution despite the heterogeneity of the underlying data collection and storage systems. An open-source analysis software package was developed using data at 1 study site and then distributed among other sites. Each site then executed the analysis independently and without modification and the results were used to perform a meta-analysis with a random-effects model.

### Data Sources

We used data from 8 sources in 3 countries, comprising data from multiple health care systems. The sources were Truven MarketScan Commercial Claims and Encounters; Columbia University Medical Center; IQVIA Disease Analyzer France; Truven MarketScan Medicare; Mount Sinai Icahn School of Medicine; Optum Clinformatics Data Mart; Ajou University School of Medicine, South Korea; and Stanford University. Four sources are EMRs from academic medical centers (Stanford, Mount Sinai, Ajou, and Columbia), 1 source is EMRs from France, and 3 sources are from nationwide medical claims in the United States (Truven MarketScan Medicare, Truven MarketScan Commercial Claims and Encounters, and Optum).

Data at each site were transformed into the Observational Medical Outcomes Partnership Common Data Model (OMOP-CDM) schema.^14^ The OMOP-CDM unifies data from heterogeneous EMRs and medical insurance claims sources with respect to terminologies and overall structure, allowing us to incorporate data from multiple health care systems around the world into our analysis. Each site obtained institutional review board approval for the analysis, or used deidentified data and thus the analysis was determined not to be human subjects research and informed consent was not deemed necessary at any site. The characteristics of the data sets from each site are summarized in Table 1. We followed the Strengthening the Reporting of Observational Studies in Epidemiology (STROBE) reporting guidelines in reporting our results.^24^

**Table 1.** Patient-Level Characteristics Across Data Sources

Data Source	No. of Patients	%		Time, y		
		Female	Male	Start	End	Total
Truven MarketScan Commercial Claims and Encounters	135 249 219	51.1	48.2	2000	2017	7
Columbia University Medical Center	5 405 830	55.9	43.7	1985	2016	31
IQVIA Disease Analyzer France	9 949 909	52.3	47.1	1997	2016	19
Truven MarketScan Medicare Supplemental and Coordination of Benefits	9 825 381	55.3	44.6	2000	2017	7
Mount Sinai	1 941 454	56.1	43.7	1979	2014	35
Optum Clinformatics Data Mart	79 604 449	50.5	49.4	2000	2017	7
Ajou University School of Medicine, South Korea	2 275 118	48.0	52.0	1994	2015	21
Stanford Health Care	2 307 445	54.3	45.4	2007	2017	10
Total No. of patients	246 558 805	51.5	48.5			

### Conversion of Data to the OMOP-CDM

The OMOP-CDM structures and harmonizes patient-level data including details of visits with health care services, diagnoses, medical procedures performed, drugs prescribed, laboratory tests and their results, and deidentified clinical note content. This is achieved by adopting common conventions for representing these records (eg, a diagnosis record consists of a patient identifier, the date of diagnosis, and a code for the diagnosis itself) across all sites, and mapping coding systems used at individual sites (eg, *International Classification of Diseases, Ninth Revision, Clinical Modification*, *International Classification of Diseases, Tenth Revision*, *International Classification of Diseases, Tenth Revision, Clinical Modification*, *Current Procedural Terminology*, fourth edition) to the OMOP-CDM Standardized Vocabularies.^15^ In this mapping process, the Systematic Nomenclature of Medicine (SNOMED) is used as the target vocabulary for diagnosis codes, RxNorm for drugs, and Logical Observation Identifiers Names and Codes for other observations such as laboratory tests and vitals measurements. Procedure codes that are in *International Classification of Diseases, Ninth Revision* are mapped to SNOMED, and *Current Procedural Terminology* codes are kept as is as part of the OMOP-CDM Standardized Vocabularies. As a result, a query using the SNOMED concept 201826 for T2D would retrieve records where a patient had an *International Classification of Diseases, Ninth Revision, Clinical Modification* or *International Classification of Diseases, Tenth Revision, Clinical Modification *code corresponding to this concept. We used age, sex, all medications, diagnoses, and procedures that were reported in the medical records of patients in the treatment and comparator groups. The propensity model and outcome definitions all operate on data that are converted into the common data model.

Each site participating in this study managed the mapping of its individual coding systems to the OMOP-CDM Standardized Vocabularies. Best practices developed by members of the OHDSI community are shared publicly to reduce variation in mapping (https://github.com/OHDSI/Themis). Additional details on the design principles of the common data model are described in the eAppendix in the Supplement.

### Cohort Construction

We used specific combinations of drugs, diagnosis codes, and laboratory test values to identify patients with T2D who received a second-line treatment. A visual explanation of cohort construction is provided in eFigure 1 in the Supplement. Briefly, a patient was included in the study if his or her medical record had a metformin prescription with a prior mention of a T2D code; no prior prescriptions of a second-line drug including insulin; no prior mentions of type 1 diabetes codes; hemoglobin A_1c _(HbA_1c_) laboratory measurements both before and after metformin prescription; and subsequent prescription of a second-line drug at least 90 days after the metformin prescription. We limited our analysis to the 3 second-line treatment categories: sulfonylureas, DPP-4 inhibitors, and thiazolidinediones for which we had enough patient data across all sites.

### Outcomes

Our primary outcome was the first observation of an HbA_1c_ level of 7% of total hemoglobin or less (to convert to proportion of total hemoglobin, multiply by 0.01) after prescription of the second-line drug, which is the goal of pharmacotherapy in most settings.^6^ We also examined several secondary outcomes: the first occurrences of myocardial infarction, kidney disorders, and eye disorders. We discerned the occurrence of these outcomes using HbA_1c_ laboratory measurements and codes for the secondary outcomes. Logical Observation Identifiers Names and Codes—codes mapped to their corresponding SNOMED codes—were used to identify HbA_1c_ laboratory measurements, whereas the SNOMED codes for secondary outcomes were obtained by searching for terms in the CDM’s vocabulary tables. A detailed list of codes representing myocardial infarction, kidney disorders, and eye disorders used in this study is provided in eTable 1 in the Supplement.

### Statistical Analysis

Three second-line treatment options after initial prescription of metformin were considered: sulfonylureas, DPP-4 inhibitors, or thiazolidinediones. We thus performed 3 pairwise comparisons: sulfonylureas vs DPP-4 inhibitors; sulfonylureas vs thiazolidinediones; and DPP-4 inhibitors vs thiazolidinediones.

We used propensity scores to mitigate biases arising from nonrandom treatment assignment at each site. For each pairwise comparison, we constructed matched cohorts using 1:1 propensity score matching with a caliper of 0.25 on the logit scale.^16,17^ The propensity scores were estimated by L1 regularized logistic regression, tuned by 10-fold cross validation, using the Cyclops package (https://github.com/ohdsi/cyclops). The propensity score models used the presence or absence of all recorded drug prescriptions, disease diagnoses, and procedures in the year prior to the index date as independent variables associated with the second-line treatment (Figure 1G). To avoid bias, no posttreatment measurements were used for matching.^18^

We then fit a Cox proportional hazard model to the matched cohorts using the CohortMethod R package (https://github.com/OHDSI/CohortMethod) and calculated the hazard ratio (HR) for each of the outcomes of interest, along with associated 95% confidence intervals. Performing an outcome regression after matching has been shown to reduce residual bias and variance.^19^ Note, that some patients were exposed to a third-line treatment, distinct from and subsequent to the second-line treatment. In these cases, we considered the patient to be right-censored at the time of prescription of the third-line treatment. Patients were also considered censored at their last recorded time of follow-up.

Propensity score matching and regression effectively remove measured confounding but cannot adjust for unmeasured confounding or measurement errors, which must be addressed separately.^20^ Manual medical record review to identify measurement error is not possible at the scale of our study, nor does it identify unmeasured confounding, which may also differ across sites. To address these issues at scale, we empirically calibrated our results using negative control outcomes.^21^ A negative control outcome is an outcome that, to our knowledge, does not have association with the exposures of interest. The fraction of negative controls that end up as associated estimates the chance of our association of interest (ie, the study question) being deemed present even if no association exists in reality. We used a set of 43 negative control outcomes (eTable 2 in the Supplement), for which we had enough data, and reapplied our analysis pipeline to estimate the associations between each exposure and these negative control outcomes. Doing so produced effect estimates (all of which are null in truth) that we used to recalibrate the *P* value for our true outcomes of interest using the methods by Schuemie and colleagues.^22^ Using negative controls, the *P* values for the HRs estimated from the Cox proportional hazard models were empirically calibrated at each study site by using the EmpericalCalibration package implemented in R (https://github.com/OHDSI/EmpiricalCalibration).

We implemented the analysis pipeline, including cohort definition and extraction, matching, calculation of HR, and empirical calibration of *P* values in the R statistical programming environment^23^ in the form of the DiabetesTxPath R Package (https://github.com/rohit43/DiabetesTxPath). The R package was then shared with other sites participating in the study and executed independently at each site without modification. Identical replication corrects for site-specific measured confounding via independent propensity score models and addresses other site-specific biases via empirical calibration. The HR of each outcome from each study site was obtained and meta-analyzed using a random-effects model to quantify a consensus HR for each second-line therapy comparison and outcome, using the meta R package (R 3.4.3 Kite-Eating Tree).

## Results

### Patient Population

Data from 246 558 805 patients (126 977 785 were female [51.5%]) spanning over 8 data sources in 3 countries were considered for this analysis. eTable 3 in the Supplement shows the total number of patients in the cohort used for the HbA_1c_ outcome analysis, for each pairwise comparison and in each data source, before and after matching. Similarly, the number of patients before and after matching for each drug comparison across the data sources for secondary outcomes (myocardial infarction, kidney disorders, and eye disorders) is provided in eTables 4 through 6 in the Supplement. Detailed information related to patient age for each drug and outcome comparison across all the 8 study sites is provided in eTables 7 through 14 in the Supplement. The mean values of HbA_1c_ before and after index date in each cohort are provided in eTables 15 through 17 in the Supplement.

### Comparative Effectiveness of Second-Line Treatments for T2D

**Figure 2.**
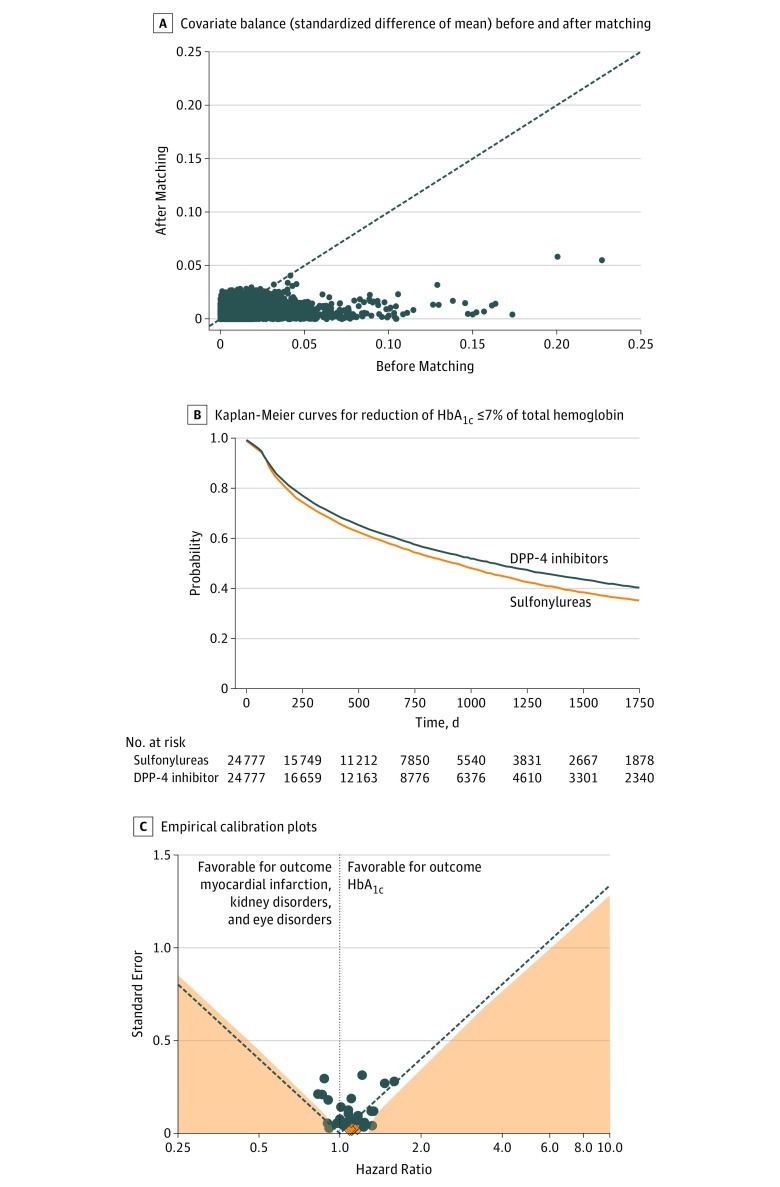
Comparative Effectiveness of Sulfonylureas vs Dipeptidyl Peptidase 4 (DPP-4) Inhibitors Using Data From Optum Clinformatics Data Mart A, Covariate balance (standardized difference of means) before and after matching. B, Kaplan-Meier curves for reduction of HbA_1c_ (HbA_1c_) to 7% of total hemoglobin or less (to convert to proportion of total hemoglobin, multiply by 0.01). C, Empirical calibration plots where estimates below the dashed line have *P* < .05 using traditional *P* value calculation. Estimates in the light orange area have *P* < .05 using calibrated *P* value calculation. Dark orange diamonds represents outcome and blue dots represent negative controls. T2D indicates type 2 diabetes.

**Figure 3.**
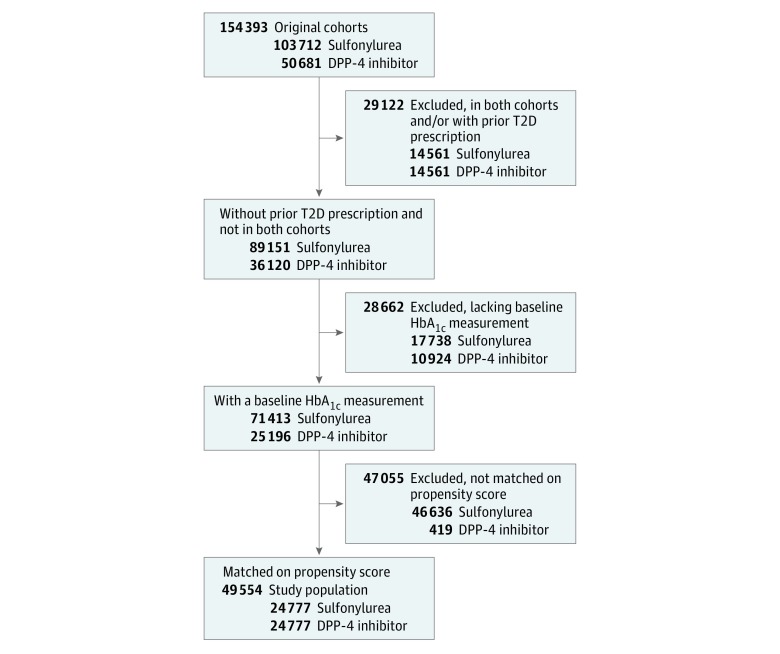
Flowchart of Matched Cohort Construction The treatment cohort included sulfonylureas and the comparator cohort included dipeptidyl peptidase 4 (DPP-4) inhibitors.

We compared the association of T2D second-line treatments with the outcome of reaching HbA_1c_ levels of 7% of total hemoglobin or less and with secondary adverse outcomes (myocardial infarction, kidney disorders, and eye disorders). Our approach is summarized in Figure 2, which shows the comparison of sulfonylureas vs DPP-4 inhibitors using data from Optum Clinformatics Data Mart. The unmatched cohort comprised 103 712 patients who received a sulfonylurea as second-line treatment vs 50 681 patients who received a DPP-4 inhibitor. After excluding 17 738 patients from the sulfonylureas group and 10 924 patients from the DPP-4 inhibitors group who were lacking baseline HbA_1c_ measurements, we were left with 71 413 and 25 196 patients in the sulfonylureas and DPP-4 inhibitors treatment groups, respectively. After 1:1 propensity score matching using pretreatment drug prescriptions, disease diagnosis, procedure, and demographics as covariates, we obtained a cohort with 24 777 patients in each treatment group (Figure 3). The covariate balance achieved after matching is illustrated as the standardized mean difference in Figure 2A.

**Figure 4.**
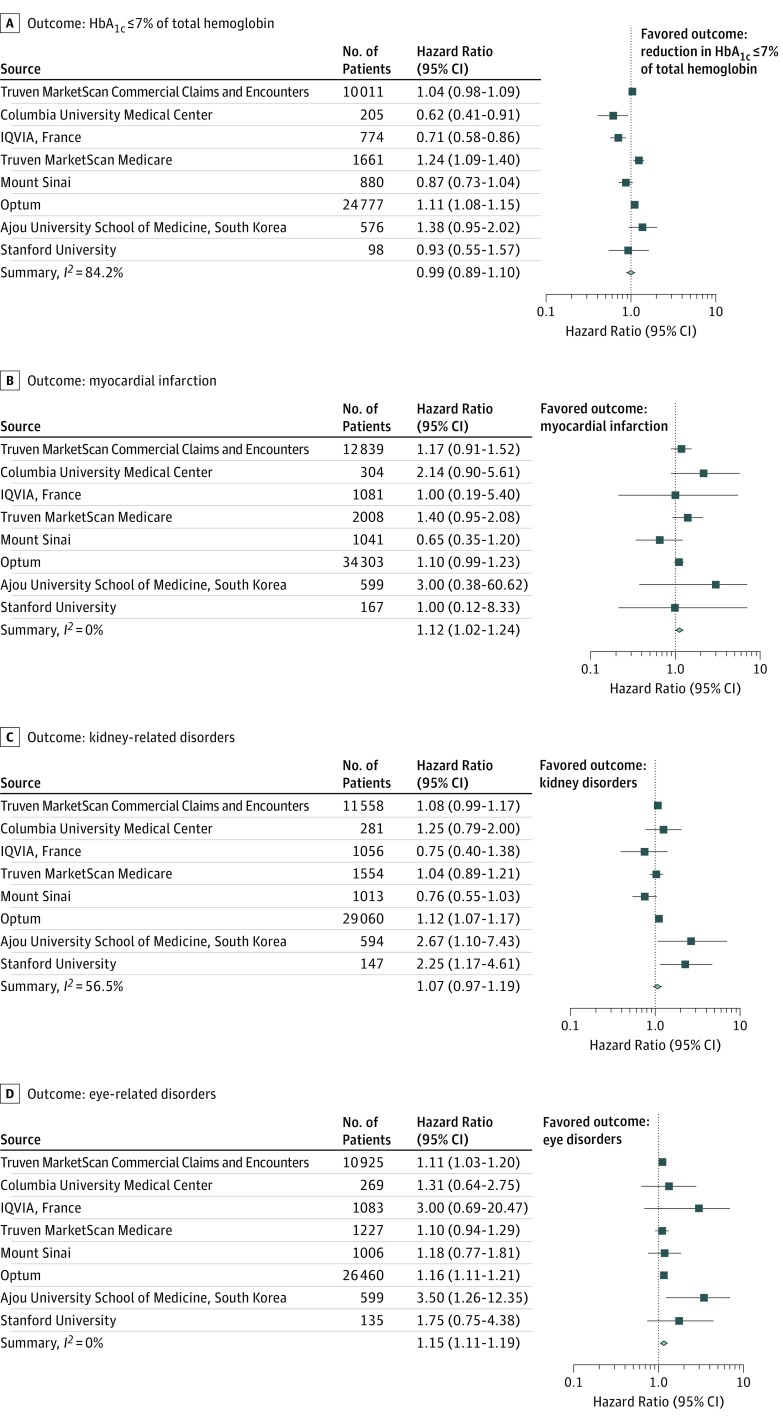
Estimated and Consensus Hazard Ratios for the Comparative Effectiveness and Safety of Sulfonylureas vs Dipeptidyl Peptidase 4 Inhibitors A, Hazard ratio for reaching a hemoglobin A_1c _(HbA_1c_) level of 7% of total hemoglobin or less (to convert to proportion of total hemoglobin, multiply by 0.01) after treatment with sulfonylureas compared with dipeptidyl peptidase 4 inhibitors. The consensus effect (summary) is based on meta-analysis of site-specific estimates. A hazard ratio greater than 1 implies sulfonylureas are associated with a higher hazard of reaching HbA_1c_ of 7% of total hemoglobin or less compared with dipeptidyl peptidase 4 inhibitors. B-D, Hazard ratios of myocardial infarction (B), kidney disorders (C), and eye disorders (D). A hazard ratio greater than 1 implies sulfonylureas have higher hazard of that outcome compared with dipeptidyl peptidase 4 inhibitors. The *I*^2^ values for each meta-analysis are shown in the bottom left of each outcome box.

The HR in the matched cohort was calculated using a Cox proportional hazard model for each of the outcomes of interest (Figure 4). The same analysis for each of the 3 comparisons and the 4 outcomes was carried out at each study site. The HR estimates were then synthesized into a consensus HR estimate using a random-effects model. For the primary outcome, the uncalibrated results from Optum Clinformatics Data Mart shows that patients who received sulfonylureas had increased hazard of a reduction in their HbA_1c_ levels as compared with those who received DPP-4 inhibitors (HR, 1.11; 95% CI, 1.08-1.15) (Figure 4A). However, on calibration of the *P* value using negative controls, we obtained a *P* value of .81, indicating that the observed hazard ratio is not significant even though the traditional *P* value indicates significance. Different sites show different HRs as seen in Truven MarketScan Medicare (HR, 1.24; 95% CI, 1.09-1.40), Columbia University Medical Center (HR, 0.62; 95% CI, 0.41-0.91), and IQVIA Disease Analyzer France (HR, 0.71; 95% CI, 0.58-0.86) for the same comparison (Figure 4A and eTable 18 in the Supplement). On calibration using negative controls, in 3 of 8 sources, the recalibrated *P* values indicated that the observed effect sizes were not significant (eTable 18 in the Supplement). Finally, given the study heterogeneity, we performed a random-effects meta-analysis across all the data sets. This meta-analysis indicated that there was not a significant difference between sulfonylureas vs DPP-4 inhibitors in the reduction of HbA_1c_ levels to 7% of total hemoglobin or less (consensus HR, 0.99; 95% CI, 0.89-1.10) (Table 2 and Figure 4A).

**Table 2.** Consensus Hazard Ratio Estimates for Primary and Secondary Outcomes After Meta-analysis^a^

Outcome	Consensus Hazard Ratio (95% CI)		
	Sulfonylureas (T) vs DPP-4 Inhibitors (C)	Sulfonylureas (T) vs Thiazolidinediones (C)	DPP-4 Inhibitors (T) vs Thiazolidinediones (C)
Reduction of HbA_1c_ to ≤7% of total hemoglobin	0.99 (0.89-1.10)	1.06 (0.96-1.16)	1.08 (0.96-1.21)
Myocardial infarction	1.12 (1.02-1.24)	1.07 (0.92-1.24)	1.10 (0.96-1.25)
Kidney disorders	1.07 (0.97-1.19)	1.02 (0.91-1.13)	1.02 (0.97-1.07)
Eye disorders	1.15 (1.11-1.19)	1.05 (1.00-1.09)	0.96 (0.92-1.01)

Abbreviations: C, comparator cohort; DPP-4, dipeptidyl peptidase 4; HbA_1c_, hemoglobin A_1c_; T, treatment cohort.

SI conversion factor: To convert HbA_1c _to proportion of total hemoglobin, multiply by 0.01.

^a^ Consensus hazard ratio for the comparison of sulfonylureas vs DPP-4 inhibitors, sulfonylureas vs thiazolidinediones, and DPP-4 inhibitors vs thiazolidinediones for outcome HbA_1c_, myocardial infarction, kidney disorders, and eye disorders after meta-analysis across 8 data sources. Sulfonylureas compared with DPP-4 inhibitors were associated with slightly increased hazard of myocardial infarction and eye disorders.

For the secondary outcomes, the comparison of sulfonylureas with DPP-4 inhibitors, where study heterogeneity was low, showed a small increased hazard of myocardial infarction (consensus HR, 1.12; 95% CI, 1.02-1.24) and eye disorders (consensus HR, 1.15; 95% CI, 1.11-1.19) in the meta-analysis, although the recalibrated *P* values (eTable 18 in the Supplement) indicated that individually, at any 1 site the association was not significant (Table 2 and Figure 4B and D). No difference was observed with respect to hazard of kidney disorders (consensus HR, 1.09; 95% CI, 0.97-1.19) (Table 2 and Figure 4C).

Comparisons of sulfonylureas with thiazolidinediones, and of DPP-4 inhibitors with thiazolidinediones (Table 2; eFigures 2 and 3 in the Supplement) show no difference in reaching HbA_1c_ levels of 7% of total hemoglobin or less, or in hazard of myocardial infarction, kidney disorders, and eye disorders in patients with T2D after recalibration of *P* values as well as after the meta-analysis. The details of each drug pair comparison along with the estimated HR, confidence intervals, and calibrated *P* values are provided in eTable 18 in the Supplement.

## Discussion

Current treatment guidelines recommend metformin as the first-line treatment for T2D. However, metformin therapy may not adequately reduce HbA_1c_ levels, in which case a second-line treatment must be chosen. Despite several randomized clinical trials addressing this question,^12,13,25,26,27^ there is little consensus. Considerable variation in second-line treatments has been observed in practice,^10^ demonstrating a need for further evidence in the choice of second-line therapies for T2D.

Our meta-analysis indicates that none of the 3 drug classes (sulfonylureas, DPP-4 inhibitors, or thiazolidinediones) were preferentially associated with a reduction in HbA_1c_ levels to 7% of total hemoglobin or less. The association of second-line drugs with lowered HbA_1c_ levels varied across data sources. It is possible that differences in clinical practice, patient populations, or data standardization between study sites were in part responsible for this site-to-site variation.

We did not observe a significant difference in secondary outcomes when comparing sulfonylureas with thiazolidinediones or DPP-4 inhibitors with thiazolidinediones. We observed that patients receiving sulfonylureas had a small increased hazard of myocardial infarction and eye disorders when compared with patients receiving DPP-4 inhibitors in the meta-analysis. However, the effect size is small. Our findings support preferring DPP-4 inhibitors over sulfonylureas as second-line therapies, in agreement with the February 2017 recommendation from the American Association of Clinical Endocrinologists and American College of Endocrinology, which did not inform our study given the timing and the date ranges of the data sets used.^7^

The OHDSI collaborative aims to translate methods research and insights into a suite of applications and exploration tools that enable the ultimate goal of generating evidence about all aspects of health care to serve the needs of patients, clinicians, and other decision makers around the world. Our study was limited to 8 data sources but the analysis could be executed at other sites that have adopted the OMOP-CDM. By allowing the study to extend to additional sites, and periodically rerunning the study, we can obtain a live estimate as part of a learning health care system.

### Limitations

Our study had limitations. The first set of limitations arises from data quality issues inherent to working with large health care databases: covariates, exposures, and outcomes may be inadequately or incorrectly measured. Data standardization into a common data model, propensity score matching, calibration via negative controls, and meta-analysis all help protect from making erroneous conclusions.

Despite standardization of data across the OHDSI network, we were unable to include laboratory values or temporal information (ie, when a variable was measured in the patient’s timeline) in the propensity score models. We accounted for this by using a large number of covariates, increasing the possibility of discovering good proxies. For example, if chronic kidney disease was present for a patient but not coded, it was still possible for the propensity score model to rely on increased creatinine laboratory orders. Fitting separate propensity models at each site allowed finding the most relevant proxies at each site, when necessary. However, it is possible that some confounders (eg, social determinants of health) have few adequate proxies captured in EMRs. Calibrating with negative control outcomes allowed us to empirically quantify the effect of confounding and systematic biases. However, despite all of our efforts, there may have remained some important confounders that were unmeasured, did not have good proxies, and were not surfaced by negative controls.

It is also possible that there were errors in the measurement of the exposure or outcomes. Although misclassification of drug prescriptions was extremely unlikely, it is possible that not all patients who were exposed to each drug were included in our study or included at the time of their first exposure. This would affect our results if the unrecorded prescriptions were not random (eg, we missed women more often than men). Calibration using negative control outcomes helped protect from exposure-related biases since those biases would also have affected the effect estimates for the negative controls. Measurement errors in outcomes of interest could also have biased our result. This would have occurred if the measurement errors (eg, missed measurements) were systematically different between treatment groups, which is unlikely in this setting for our primary outcome. For instance, because the laboratory test is standardized, there is no reason that HbA_1c_ measurements would have been lower just for patients receiving DPP-4 inhibitors than for patients receiving sulfonylureas. An important outcome that we did not examine is hypoglycemia, which is difficult to reliably ascertain in the data we have.

Another set of limitations concern the study design rather than the data. Because of our matching procedure, our results apply only to patients who were at equipoise and likely to receive either treatment. Patients who were very likely to receive a particular treatment were discarded in matching. We did not assess whether metformin was titrated up to maximal dose; instead, we relied on the fact that a second-line drug was prescribed after at least 90 days of initial prescription of metformin, suggesting metformin was ineffective for a patient to control HbA_1c_, or possibly resulted in adverse effects. We also did not account for the dose levels of the second-line drugs because of the difficulty of accurately estimating dose-response in observational data. However, the wide use of existing diabetes treatment guidelines ensures that dosing was generally standardized.

There is evidence of considerable heterogeneity of effects among the study sites for our primary outcome of HbA_1c_ reduction. Our random-effects meta-analysis averaged over these differences and would fail to detect an effect. In studies using large data—where there is a risk of seeing spurious associations—it is more important to not be wrong in declaring an association than to try to detect every association that exists. While elucidating the sources of this heterogeneity is beyond the scope of this current work, performing such studies via a collaborative research network with a shared study design eliminates heterogeneity owing to study design choices and surfaces between site disagreements in a high-throughput, empirical manner. In some cases, doing so might uncover true treatment effect heterogeneity. In cases where there is less evidence of such heterogeneity, such as our secondary outcomes, meta-analysis allowed us to increase power and precision beyond what is possible at a single-study site.

## Conclusions

Two-way comparisons among DPP-4, sulfonylureas, and thiazolidinediones for a difference in lowering HbA_1c_ levels to 7% of total hemoglobin or less in patients with T2D treated with metformin as a first-line therapy were inconclusive after meta-analysis as well as after empirical calibration. Our study is an example of a large multinational study in an open collaborative research network, made feasible via the adoption of a common data model and open-source analytical tools. By taking advantage of this standardization, we were able to develop an open, reusable analysis pipeline that enabled large-scale characterization of the effectiveness of T2D therapy across nations.

## Notes

Supplement. **eAppendix.** OMOP Common Data Model **eTable 1.** Concept IDs Utilized for Outcome MI, KD, ED and KD **eTable 2.** Concepts Used as Negative Controls for *P* Value Calibration **eTable 3.** Number of Patients Before and After Matching for Each Drug Comparison and Outcome HbA_1c_ **eTable 4.** Number of Patients Before and After Matching for Each Drug Comparison and Outcome Myocardial Infarction **eTable 5.** Number of Patients Before and After Matching for Each Drug Comparison and Outcome Kidney Disorders **eTable 6.** Number of Patients Before and After Matching for Each Drug Comparison and Outcome Eye Disorders **eTable 7.** Age Information Before and After Matching for Each Drug Comparison Based on the Data From Truven MarketScan CCAE **eTable 8.** Age Information Before and After Matching for Each Drug Comparison Based on the Data From Columbia University **eTable 9.** Age Information Before and After Matching for Each Drug Comparison Based on the Data From IQVIA Disease Analyzer France **eTable 10.** Age Information Before and After Matching for Each Drug Comparison Based on the Data From Truven MarketScan MDCR **eTable 11.** Age Information Before and After Matching for Each Drug Comparison Based on the Data From Mount Sinai **eTable 12.** Age Information Before and After Matching for Each Drug Comparison Based on the Data From Optum Clinformatics Data Mart **eTable 13.** Age Information Before and After Matching for Each Drug Comparison Based on the Data From Ajou University, South Korea **eTable 14.** Age Information Before and After Matching for Each Drug Comparison Based on the Data From Stanford University **eTable 15.** Mean, Median and Standard Deviation of HbA_1c_ Values for the Comparison of Sulfonylureas vs DPP4 Inhibitors Across Eight Study Sites **eTable 16.** Mean, Median and Standard Deviation of HbA_1c_ Values for the Comparison of Sulfonylureas vs Thiazolidinediones Across Eight Study Sites **eTable 17.** Mean, Median and Standard Deviation of HbA_1c_ Values for the Comparison of DPP4 Inhibitors vs Thiazolidinediones Across Eight Study Sites **eTable 18.** Number of Patients, Hazard Ratio, Confidence Intervals (CI), *P* Values and Calibrated *P* Values for Each Drug Comparison and Each Outcome Based on Analysis Across All Eight Study Sites **eFigure 1.** Cohort Construction **eFigure 2.** Comparative Effectiveness of Sulfonylureas vs Thiazolidinediones **eFigure 3.** Comparative Effectiveness of DPP-4 Inhibitors vs Thiazolidinediones
