# Supplementary material for: Association of Hemoglobin A1c Levels With Use of Sulfonylureas, Dipeptidyl Peptidase 4 Inhibitors, and Thiazolidinediones in Patients With Type 2 Diabetes Treated With Metformin: Analysis From the Observational Health Data Sciences and Informatics Initiative
Source: JAMA Netw Open. 2018 Aug 24;1(4):e181755. doi: 10.1001/jamanetworkopen.2018.1755 (PMC6324274; doi:10.1001/jamanetworkopen.2018.1755)
Supplement: Supplement. — eAppendix. OMOP Common Data Model eTable 1. Concept IDs Utilized for Outcome MI, KD, ED and KD eTable 2. Concepts Used as Negative Controls for P Value Calibration eTable 3. Number of Patients Before and After Matching for Each Drug Comparison and Outcome HbA1c eTable 4. Number of Patients Before and After Matching for Each Drug Comparison and Outcome Myocardial Infarction eTable 5. Number of Patients Before and After Matching for Each Drug Comparison and Outcome Kidney Disorders eTable 6. Number of Patients Before and After Matching for Each Drug Comparison and Outcome Eye Disorders eTable 7. Age Information Before and After Matching for Each Drug Comparison Based on the Data From Truven MarketScan CCAE eTable 8. Age Information Before and After Matching for Each Drug Comparison Based on the Data From Columbia University eTable 9. Age Information Before and After Matching for Each Drug Comparison Based on the Data From IQVIA Disease Analyzer France eTable 10. Age Information Before and After Matching for Each Drug Comparison Based on the Data From Truven MarketScan MDCR eTable 11. Age Information Before and After Matching for Each Drug Comparison Based on the Data From Mount Sinai eTable 12. Age Information Before and After Matching for Each Drug Comparison Based on the Data From Optum Clinformatics Data Mart eTable 13. Age Information Before and After Matching for Each Drug Comparison Based on the Data From Ajou University, South Korea eTable 14. Age Information Before and After Matching for Each Drug Comparison Based on the Data From Stanford University eTable 15. Mean, Median and Standard Deviation of HbA1c Values for the Comparison of Sulfonylureas vs DPP4 Inhibitors Across Eight Study Sites eTable 16. Mean, Median and Standard Deviation of HbA1c Values for the Comparison of Sulfonylureas vs Thiazolidinediones Across Eight Study Sites eTable 17. Mean, Median and Standard Deviation of HbA1c Values for the Comparison of DPP4 Inhibitors vs Thiazolidinediones Acros [file jamanetwopen-1-e181755-s001.pdf]

Supplementary Online Content

Vashisht R, Jung K, Schuler A, et al. Association of hemoglobin A_{1c} levels with use of sulfonylureas, dipeptidyl peptidase 4 inhibitors, and thiazolidinediones in patients with type 2 diabetes treated with metformin: analysis from the Observational Health Data Sciences and Informatics Initiative. *JAMA Netw Open*. 2018;1(4):e181755. doi:10.1001/jamanetworkopen.2018.1755

eAppendix. OMOP Common Data Model
eTable 1. Concept IDs Utilized for Outcome MI, KD, ED and KD
eTable 2. Concepts Used as Negative Controls for *P* Value Calibration
eTable 3. Number of Patients Before and After Matching for Each Drug Comparison and Outcome HbA_{1c}
eTable 4. Number of Patients Before and After Matching for Each Drug Comparison and Outcome Myocardial Infarction
eTable 5. Number of Patients Before and After Matching for Each Drug Comparison and Outcome Kidney Disorders
eTable 6. Number of Patients Before and After Matching for Each Drug Comparison and Outcome Eye Disorders
eTable 7. Age Information Before and After Matching for Each Drug Comparison Based on the Data From Truven MarketScan CCAE
eTable 8. Age Information Before and After Matching for Each Drug Comparison Based on the Data From Columbia University
eTable 9. Age Information Before and After Matching for Each Drug Comparison Based on the Data From IQVIA Disease Analyzer France
eTable 10. Age Information Before and After Matching for Each Drug Comparison Based on the Data From Truven MarketScan MDCR
eTable 11. Age Information Before and After Matching for Each Drug Comparison Based on the Data From Mount Sinai
eTable 12. Age Information Before and After Matching for Each Drug Comparison Based on the Data From Optum Clinformatics Data Mart
eTable 13. Age Information Before and After Matching for Each Drug Comparison Based on the Data From Ajou University, South Korea
eTable 14. Age Information Before and After Matching for Each Drug Comparison Based on the Data From Stanford University
eTable 15. Mean, Median and Standard Deviation of HbA_{1c} Values for the Comparison of Sulfonylureas vs DPP4 Inhibitors Across Eight Study Sites
eTable 16. Mean, Median and Standard Deviation of HbA_{1c} Values for the Comparison of Sulfonylureas vs Thiazolidinediones Across Eight Study Sites
eTable 17. Mean, Median and Standard Deviation of HbA_{1c} Values for the Comparison of DPP4 Inhibitors vs Thiazolidinediones Across Eight Study Sites

© 2018 Vashisht R et al. *JAMA Network Open*.

eTable 18. Number of Patients, Hazard Ratio, Confidence Intervals (CI), *P* Values and Calibrated *P* Values for Each Drug Comparison and Each Outcome Based on Analysis Across All Eight Study Sites
eFigure 1. Cohort Construction
eFigure 2. Comparative Effectiveness of Sulfonylureas vs Thiazolidinediones
eFigure 3. Comparative Effectiveness of DPP-4 Inhibitors vs Thiazolidinediones

This supplementary material has been provided by the authors to give readers additional information about their work.

© 2018 Vashisht R et al. *JAMA Network Open*.

eAppendix 1. OMOP Common Data Model

The OMOP Common Data Model (CDM) allows for the systematic analysis of disparate observational databases. The core idea is to transform data contained within different databases into a common format (data model) as well as a common representation (terminologies, vocabularies, coding schemes), and then perform systematic analyses using a library of standard analytic routines based on the common format. CDM is designed to store observational data to allow for research, under the following principles:

- **Suitability for purpose:** The CDM aims at providing data organized in a way optimal for analysis, rather than for the purpose of operational needs of health care providers or payers.
- **Data protection:** All data that might jeopardize the identity and protection of patients, such as names, precise birthdays etc. are limited. Exceptions are possible where the research expressly requires more detailed information, such as precise birth dates for the study of infants.
- **Design of domains:** The domains are modeled in a person-centric relational data model, where for each record the identity of the person and a date is captured as a minimum.
- **Rationale for domains:** Domains are identified and separately defined in an Entity-relationship model if they have an analysis use case and the domain has specific attributes that are not otherwise applicable. All other data can be preserved as an observation in an entity-attribute-value structure.
- **Standardized Vocabularies:** To standardize the content of those records, the CDM relies on the Standardized Vocabularies containing all necessary and appropriate corresponding standard healthcare concepts.
- **Reuse of existing vocabularies:** If possible, these concepts are leveraged from national or industry standardization or vocabulary definition organizations or initiatives, such as the National Library of Medicine, the Department of Veterans' Affairs, the Center of Disease Control and Prevention, etc.
- **Maintaining source codes:** Even though all codes are mapped to the Standardized Vocabularies, the model also stores the original source code to ensure no information is lost.
- **Technology neutrality:** The CDM does not require a specific technology. It can be realized in any relational database, such as Oracle, SQL Server etc., or as SAS analytical datasets.
- **Scalability:** The CDM is optimized for data processing and computational analysis to accommodate data sources that vary in size, including databases with up to hundreds of millions of persons and billions of clinical observations.
- **Backwards compatibility:** All changes from previous CDMs are clearly delineated. Older versions of the CDM can be easily created from this CDMv5, and no information is lost that was present previously.

© 2018 Vashisht R et al. *JAMA Network Open*.

Detailed discussion related to the above mentioned design principles of OMOP-CDM data model can be explored in detail at https://github.com/OHDSI/CommonDataModel/wiki

© 2018 Vashisht R et al. *JAMA Network Open*.

eTable 1. Concept IDs Utilized for Outcome MI, KD, ED and KD

**Comparison:** Sulfonylureas vs DPP4 inhibitors **– Outcome:** Myocardial Infarction			
**Drug**	**Concept Identifier**	**Concept Name**	**Number of Patients**
**Sulfonylureas**	312327	Acute myocardial infarction	3
	438170	Acute myocardial infarction of inferior wall	1
**DPP4 inhibitors**	444406	Acute subendocardial infarction	2
	312327	Acute myocardial infarction	1
**Comparison:** Sulfonylureas vs DPP4 inhibitors **– Outcome:** Kidney Disorders			
**Drug**	**Concept Identifier**	**Concept Name**	**Number of Patients**
**Sulfonylureas**	443731	Renal disorder due to type 2 diabetes mellitus	14
	192279	Diabetic renal disease	11
	197320	Acute renal failure syndrome	9
	201620	Kidney stone	4
	198185	Chronic renal failure	3
	439697	Hypertensive renal disease with renal failure	3
	443597	Chronic kidney disease stage 3	3
	198199	Pyelonephritis	2
	439696	Hypertensive heart and renal disease with (congestive) heart failure	2
	443601	Chronic kidney disease stage 2	2
	193016	Cystic disease of kidney	1
	193253	Nephritis	1
	194152	Renal agenesis and dysgenesis	1
	194686	Acquired renal cystic disease	1
	198985	Primary malignant neoplasm of kidney	1
	433811	Hydronephrosis	1
**DPP4 inhibitors**	197320	Acute renal failure syndrome	6
	192279	Diabetic renal disease	2
	193016	Cystic disease of kidney	2
	201620	Kidney stone	2
	443731	Renal disorder due to type 2 diabetes mellitus	2
	444044	Acute tubular necrosis	2
	192359	Renal failure syndrome	1
	193253	Nephritis	1
	194686	Acquired renal cystic disease	1

© 2018 Vashisht R et al. *JAMA Network Open*.

	198185	Chronic renal failure	1
	433811	Hydronephrosis	1
	439697	Hypertensive renal disease with renal failure	1
	443597	Chronic kidney disease stage 3	1
**Comparison:** Sulfonylureas vs DPP4 inhibitors **– Outcome:** Eye Disorders			
**Drug**	**Concept Identifier**	**Concept Name**	**Number of Patients**
**Sulfonylureas**	439297	Nuclear senile cataract	10
	376683	Nonproliferative diabetic retinopathy	4
	4102183	Borderline glaucoma	2
	375545	Cataract	1
	377552	Moderate nonproliferative diabetic retinopathy	1
	378743	Mild non-proliferative diabetic retinopathy	1
	432311	Angle-closure glaucoma - borderline	1
	435810	Capsular fibrosis	1
	437541	Glaucoma	1
**DPP4 inhibitors**	439297	Nuclear senile cataract	6
	381295	Age-related cataract	2
	372894	Central serous chorioretinopathy	1
	375545	Cataract	1
	376683	Nonproliferative diabetic retinopathy	1
	435262	Primary open angle glaucoma	1
	4102183	Borderline glaucoma	1
**Comparison:** Sulfonylureas vs Thiazolidinediones **– Outcome:** Myocardial Infarction			
**Drug**	**Concept Identifier**	**Concept Name**	**Number of Patients**
**Sulfonylureas**	438170	Acute myocardial infarction of inferior wall	1
**Thiazolidinediones**	444406	Acute subendocardial infarction	2
**Comparison:** Sulfonylureas vs Thiazolidinediones **– Outcome:** Kidney disorders			
**Drug**	**Concept Identifier**	**Concept Name**	**Number of Patients**
**Sulfonylureas**	443731	Renal disorder due to type 2 diabetes mellitus	9
	197320	Acute renal failure syndrome	8
	192279	Diabetic renal disease	7
	443597	Chronic kidney disease stage 3	6
	439697	Hypertensive renal disease with renal failure	5
	198185	Chronic renal failure	3

© 2018 Vashisht R et al. *JAMA Network Open*.

	193253	Nephritis	2
	201620	Kidney stone	2
	443614	Chronic kidney disease stage 1	2
	195314	Nephrotic syndrome	1
	198199	Pyelonephritis	1
	433811	Hydronephrosis	1
	444044	Acute tubular necrosis	1
**Thiazolidinediones**	192279	Diabetic renal disease	5
	443731	Renal disorder due to type 2 diabetes mellitus	5
	197320	Acute renal failure syndrome	4
	201620	Kidney stone	2
	439697	Hypertensive renal disease with renal failure	2
	443597	Chronic kidney disease stage 3	2
	443601	Chronic kidney disease stage 2	2
	193016	Cystic disease of kidney	1
	193782	End stage renal disease	1
	198185	Chronic renal failure	1
**Comparison:** Sulfonylureas vs Thiazolidinediones **– Outcome:** Eye disorder			
**Drug**	**Concept Identifier**	**Concept Name**	**Number of Patients**
**Sulfonylureas**	439297	Nuclear senile cataract	10
	375545	Cataract	4
	380097	Diabetic macular edema	2
	435262	Primary open angle glaucoma	2
	437541	Glaucoma	2
	376114	Severe nonproliferative diabetic retinopathy	1
	376683	Nonproliferative diabetic retinopathy	1
	376965	Hypertensive retinopathy	1
	380096	Proliferative diabetic retinopathy	1
	432626	Glaucoma associated with vascular disorder	1
	4102183	Borderline glaucoma	1
**Thiazolidinediones**	439297	Nuclear senile cataract	7
	375545	Cataract	3
	378743	Mild non-proliferative diabetic retinopathy	2
	437541	Glaucoma	2
	381295	Age-related cataract	1
	435262	Primary open angle glaucoma	1
	437851	Open angle with borderline intraocular pressure	1
	4102183	Borderline glaucoma	1

© 2018 Vashisht R et al. *JAMA Network Open*.

**Comparison:** DPP4 inhibitors vs Thiazolidinediones **– Outcome:** Myocardial Infarction			
**Drug**	**Concept Identifier**	**Concept Name**	**Number of Patients**
**DPP4 inhibitors**	444406	Acute subendocardial infarction	1
**Thiazolidinediones**	NA	NA	NA
**Comparison:** DPP4 inhibitors vs Thiazolidinediones **– Outcome:** Kidney disorders			
**Drug**	**Concept Identifier**	**Concept Name**	**Number of Patients**
**DPP4 inhibitors**	197320	Acute renal failure syndrome	6
	443731	Renal disorder due to type 2 diabetes mellitus	4
	192279	Diabetic renal disease	3
	194686	Acquired renal cystic disease	2
	443597	Chronic kidney disease stage 3	2
	444044	Acute tubular necrosis	2
	192359	Renal failure syndrome	1
	193016	Cystic disease of kidney	1
	193253	Nephritis	1
	198185	Chronic renal failure	1
	201620	Kidney stone	1
	439697	Hypertensive renal disease with renal failure	1
**Thiazolidinediones**	197320	Acute renal failure syndrome	5
	192279	Diabetic renal disease	4
	443731	Renal disorder due to type 2 diabetes mellitus	4
	198185	Chronic renal failure	3
	443601	Chronic kidney disease stage 2	3
	193016	Cystic disease of kidney	1
	201620	Kidney stone	1
	439697	Hypertensive renal disease with renal failure	1
	443597	Chronic kidney disease stage 3	1
	443961	Anemia of chronic renal failure	1
**Comparison:** DPP4 inhibitors vs Thiazolidinediones **–** **Outcome:** Eye disorders			
**Drug**	**Concept Identifier**	**Concept Name**	**Number of Patients**
**DPP4 inhibitors**	439297	Nuclear senile cataract	2
	376683	Nonproliferative diabetic retinopathy	1
	381295	Age-related cataract	1
	437541	Glaucoma	1
	4102183	Borderline glaucoma	1

© 2018 Vashisht R et al. *JAMA Network Open*.

**Thiazolidinediones**	439297	Nuclear senile cataract	6
	375545	Cataract	3
	437541	Glaucoma	3
	378743	Mild non-proliferative diabetic retinopathy	1
	381295	Age-related cataract	1
	437851	Open angle with borderline intraocular pressure	1
	4102183	Borderline glaucoma	1

SNOMED codes for secondary outcomes were obtained by searching for terms in the CDM's vocabulary tables. To build the set of SNOMED codes "eye-related disorders" and "kidney related disorders", we started with a keyword based search of SNOMED labels, and included the matching concepts as well as their subclasses in the OHDSI Standard Vocabulary (https://github.com/OHDSI/CommonDataModel/wiki/Standardized-Vocabularies).

This process generates a seemingly large list of concepts that correspond to the outcome of interest. In practice, not all the child codes are instantiated in the data. We show the frequency at which specific codes were actually seen in the cohorts analyzed at Stanford to illustrate which codes are actually instantiated in the data. Concepts utilized for identifying patients with myocardial Infarction, kidney disorders and eye disorders are shown in the table below. Each concept is ordered based on the total number of patients identified by the concept.

eTable 2. Concepts Used As Negative Controls for *P* Value Calibration

**Concept Identifier**	**Concept Name**
376707	Acute conjunctivitis
433753	Alcohol abuse
257007	Allergic rhinitis
442077	Anxiety disorder
436665	Bipolar disorder
380094	Carpal tunnel syndrome
255573	Chronic obstructive lung disease
257012	Chronic sinusitis
443617	Conduct disorder
134438	Contact dermatitis
78619	Contusion of knee
378752	Corneal opacity
137063	Corns and callus
133228	Dental caries
134681	Diffuse spasm of esophagus
432251	Disease caused by parasite
378161	Disorder of ear
139057	Disorder of oral soft tissues
31057	Disorder of pharynx
138225	Disorder of sebaceous gland
440329	Herpes zoster without complication
441788	Human papilloma virus infection
140673	Hypothyroidism
374375	Impacted cerumen
139099	Ingrowing nail
436962	Insomnia
201322	Internal hemorrhoids without complication
132466	Lumbar sprain
255891	Lupus erythematosus
444100	Mood disorder
440374	Obsessive-compulsive disorder
380733	Otalgia
372328	Otitis media
4002650	Plantar fasciitis
373478	Presbyopia
436073	Psychotic disorder
438688	Sarcoidosis

© 2018 Vashisht R et al. *JAMA Network Open*.

432597	Schizoaffective schizophrenia
435783	Schizophrenia
372409	Sciatica
73562	Solitary sacroiliitis
133141	Tinea pedis
436070	Vitamin D deficiency

© 2018 Vashisht R et al. *JAMA Network Open*.

eTable 3. Number of Patients Before and After Matching for Each Drug Comparison and Outcome HbA_{1c}

**Dataset**	**Sulfonylureas (T) vs DPP4 inhibitors (C)**				**Sulfonylureas (T) vs Thiazolidinediones (C)**				**DPP4 inhibitors (T) vs Thiazolidinediones (C)**			
	Unmatched		Matched		Unmatched		Matched		Unmatched		Matched	
	T	C	T	C	T	C	T	C	T	C	T	C
Truven MarketScan CCAE	3738	18962	10011	10011	37387	16945	9714	9714	18962	16945	6871	6871
Columbia University	2507	595	205	205	2507	759	269	269	595	759	175	175
IQVIA Disease Analyser France	1961	2008	774	774	1961	1020	499	499	2008	1020	389	389
Truven MarketScan MDCR	7556	2771	1661	1661	7556	2424	1440	1440	2771	2424	942	942
Mt. Sinai	2913	1862	880	880	2913	1027	417	417	1862	1027	475	475
Optum Clinformatics Data Mart	103712	50681	24777	24777	103712	43764	22953	22953	50681	43764	16763	16763
Ajou University, South Korea	1826	1330	576	576	1826	292	175	175	1330	292	205	205
Stanford University	896	275	98	98	896	256	78	78	275	256	117	117

© 2018 Vashisht R et al. *JAMA Network Open*.

eTable 4. Number of Patients Before and After Matching for Each Drug Comparison and Outcome Myocardial Infarction

**Dataset**	**Sulfonylureas (T) vs DPP4 inhibitors (C)**				**Sulfonylureas (T) vs Thiazolidinediones (C)**				**DPP4 inhibitors (T) vs Thiazolidinediones (C)**			
	Unmatched		Matched		Unmatched		Matched		Unmatched		Matched	
	T	C	T	C	T	C	T	C	T	C	T	C
Truven MarketScan CCAE	37387	18962	12839	12839	37387	16945	10887	10887	18962	16945	8197	8197
Columbia University	2507	595	304	304	2507	759	400	400	595	759	237	237
IQVIA Disease Analyser France	1961	2008	1081	1081	1961	1020	608	608	2008	1020	509	509
Truven MarketScan MDCR	7556	2771	2008	2008	7556	2424	1578	1578	2771	2424	1072	1072
Mt. Sinai	2913	1862	1041	1041	2913	1027	517	517	1862	1027	554	554
Optum Clinformatics Data Mart	103721	50681	34303	34303	103712	43764	28549	28549	50681	43764	21505	21505
Ajou University, South Korea	1826	1330	599	599	1826	292	181	181	1330	292	211	211
Stanford University	896	275	167	167	896	256	134	134	275	256	175	175

© 2018 Vashisht R et al. *JAMA Network Open*.

eTable 5. Number of Patients Before and After Matching for Each Drug Comparison and Outcome Kidney Disorders

**Dataset**	**Sulfonylureas (T) vs DPP4 inhibitors (C)**				**Sulfonylureas (T) vs Thiazolidinediones (C)**				**DPP4 inhibitors (T) vs Thiazolidinediones (C)**			
	Unmatched		Matched		Unmatched		Matched		Unmatched		Matched	
	T	C	T	C	T	C	T	C	T	C	T	C
Truven MarketScan CCAE	37387	18962	11558	11558	37387	16945	9980	9980	18962	16945	7557	7557
Columbia University	2507	595	304	304	2507	759	400	400	595	759	237	237
IQVIA Disease Analyser France	1961	2008	1056	1056	1961	1020	594	594	2008	1020	499	499
Truven MarketScan MDCR	7556	2771	1554	1554	7556	2424	1272	1272	2771	2424	888	888
Mt. Sinai	2913	1862	1013	1013	2913	1027	501	501	1862	1027	538	538
Optum Clinformatics Data Mart	103721	50681	29060	29060	103712	43764	25775	25775	50681	43764	18927	18927
Ajou University, South Korea	1826	1330	594	594	1826	292	180	180	1330	292	208	208
Stanford University	896	275	147	147	896	256	126	126	275	256	165	165

© 2018 Vashisht R et al. *JAMA Network Open*.

eTable 6. Number of Patients Before and After Matching for Each Drug Comparison and Outcome Eye Disorders

**Dataset**	**Sulfonylureas (T) vs DPP4 inhibitors (C)**				**Sulfonylureas (T) vs Thiazolidinediones (C)**				**DPP4 inhibitors (T) vs Thiazolidinediones (C)**			
	Unmatched		Matched		Unmatched		Matched		Unmatched		Matched	
	T	C	T	C	T	C	T	C	T	C	T	C
Truven MarketScan CCAE	2507	595	304	304	2507	759	400	400	595	759	237	237
Columbia University	2913	1862	1006	1006	2913	1027	472	472	1862	1027	529	529
IQVIA Disease Analyser France	1961	2008	1083	1083	1961	1020	604	604	2008	1020	508	508
Truven MarketScan MDCR	1826	1330	599	599	1826	292	179	179	1330	292	210	210
Mt. Sinai	896	275	135	135	896	256	121	121	275	256	145	145
Optum Clinformatics Data Mart	7556	2771	1227	1227	7556	2424	1063	1063	2771	2424	732	732
Ajou University, South Korea	37387	18962	10925	10925	37387	16945	9636	9636	18962	16945	7220	7220
Stanford University	103721	50681	26460	26460	103712	43764	23773	23773	50681	43764	17470	17470

© 2018 Vashisht R et al. *JAMA Network Open*.

eTable 7. Age Information Before and After Matching for Each Drug Comparison Based on the Data From Truven MarketScan CCAE

**Comparison**: Sulfonylureas vs DPP4 inhibitors - **Outcome**: HbA1c				
Age group	Unmatched		Matched	
	Sulfonylureas	DPP4 inhibitors	Sulfonylureas	DPP4 inhibitors
10-14	1	1	NA	1
15-19	20	7	4	5
20-24	109	27	17	25
25-29	279	90	63	78
30-34	823	212	186	175
35-39	1740	512	398	390
40-44	3107	1159	878	892
45-49	5020	1907	1451	1442
50-54	7113	3059	2267	2252
55-59	7721	3600	2606	2618
60-64	6095	3129	2072	2060
65-69	228	129	69	73
**Comparison**: Sulfonylureas vs DPP4 inhibitors - **Outcome**: Myocardial Infarction				
Age group	Unmatched		Matched	
	Sulfonylureas	DPP4 inhibitors	Sulfonylureas	DPP4 inhibitors
10-14	1	1	NA	1
15-19	20	7	5	7
20-24	109	27	22	27
25-29	279	90	75	90
30-34	823	212	221	208
35-39	1740	512	473	504
40-44	3107	1159	1109	1124
45-49	5020	1907	1775	1806
50-54	7113	3059	2869	2870
55-59	7721	3600	3349	3289
60-64	6095	3129	2828	2801
65-69	228	129	113	112
**Comparison**: Sulfonylureas vs DPP4 inhibitors - **Outcome**: Kidney Disorders				
Age group	Unmatched		Matched	
	Sulfonylureas	DPP4 inhibitors	Sulfonylureas	DPP4 inhibitors
10-14	1	1	NA	1
15-19	20	7	4	7
20-24	109	27	24	27
25-29	279	90	73	87
30-34	823	212	209	196
35-39	1740	512	430	460
40-44	3107	1159	1025	1036
45-49	5020	1907	1602	1667
50-54	7113	3059	2606	2610
55-59	7721	3600	3014	2922

© 2018 Vashisht R et al. *JAMA Network Open*.

60-64	6095	3129	2468	2439
65-69	228	129	103	106
**Comparison**: Sulfonylureas vs DPP4 inhibitors - **Outcome**: Eye Disorders				
Age group	Unmatched		Matched	
	Sulfonylureas	DPP4 inhibitors	Sulfonylureas	DPP4 inhibitors
10-14	1	1	NA	1
15-19	20	7	4	7
20-24	109	27	21	27
25-29	279	90	73	90
30-34	823	212	210	200
35-39	1740	512	457	474
40-44	3107	1159	1011	1037
45-49	5020	1907	1583	1645
50-54	7113	3059	2496	2478
55-59	7721	3600	2777	2744
60-64	6095	3129	2205	2131
65-69	228	129	88	91
**Comparison**: Sulfonylureas vs Thiazolidinediones - **Outcome**: HbA1c				
Age group	Unmatched		Matched	
	Sulfonylureas	Thiazolidinediones	Sulfonylureas	Thiazolidinediones
10-14	1	1	NA	1
15-19	20	9	6	7
20-24	106	24	27	21
25-29	287	67	58	62
30-34	803	262	252	232
35-39	1709	566	504	488
40-44	3014	1067	919	929
45-49	4870	1847	1663	1632
50-54	6970	2715	2301	2337
55-59	7595	2902	2491	2483
60-64	6171	1832	1451	1484
65-69	236	49	42	38
**Comparison**: Sulfonylureas vs Thiazolidinediones - **Outcome**: Myocardial Infarction				
Age group	Unmatched		Matched	
	Sulfonylureas	Thiazolidinediones	Sulfonylureas	Thiazolidinediones
10-14	1	1	NA	1
15-19	20	9	7	9
20-24	106	24	31	24
25-29	287	67	65	67
30-34	803	262	271	258
35-39	1709	566	554	557
40-44	3014	1067	1002	1033
45-49	4870	1847	1777	1774
50-54	6970	2715	2591	2584
55-59	7595	2902	2790	2753

© 2018 Vashisht R et al. *JAMA Network Open*.

60-64	6171	1832	1745	1778
65-69	236	49	54	49
**Comparison**: Sulfonylureas vs Thiazolidinediones - **Outcome**: Kidney Disorders				
Age group	Unmatched		Matched	
	Sulfonylureas	Thiazolidinediones	Sulfonylureas	Thiazolidinediones
10-14	1	1	NA	1
15-19	20	9	6	8
20-24	106	24	25	24
25-29	287	67	64	66
30-34	803	262	260	240
35-39	1709	566	503	528
40-44	3014	1067	915	962
45-49	4870	1847	1634	1675
50-54	6970	2715	2411	2365
55-59	7595	2902	2552	2500
60-64	6171	1832	1564	1568
65-69	236	49	46	43
**Comparison**: Sulfonylureas vs Thiazolidinediones - **Outcome**: Eye Disorders				
Age group	Unmatched		Matched	
	Sulfonylureas	Thiazolidinediones	Sulfonylureas	Thiazolidinediones
10-14	1	1	NA	1
15-19	20	9	5	9
20-24	106	24	32	23
25-29	287	67	68	66
30-34	803	262	258	250
35-39	1709	566	525	529
40-44	3014	1067	946	981
45-49	4870	1847	1656	1641
50-54	6970	2715	2335	2304
55-59	7595	2902	2348	2356
60-64	6171	1832	1421	1437
65-69	236	49	42	39
**Comparison**: DPP4 inhibitors vs Thiazolidinediones - **Outcome**: HbA1c				
Age group	Unmatched		Matched	
	DPP4 inhibitors	Thiazolidinediones	DPP4 inhibitors	Thiazolidinediones
10-14	1	1	1	1
15-19	9	13	4	7
20-24	32	28	22	15
25-29	115	89	57	54
30-34	273	336	166	155
35-39	636	730	354	322
40-44	1378	1379	669	659
45-49	2261	2364	1086	1084
50-54	3613	3422	1594	1614

© 2018 Vashisht R et al. *JAMA Network Open*.

55-59	4151	3534	1685	1676
60-64	3627	2260	1189	1248
65-69	135	58	44	36
**Comparison**: DPP4 inhibitors vs Thiazolidinediones - **Outcome**: Myocardial Infarction				
Age group	Unmatched		Matched	
	DPP4 inhibitors	Thiazolidinediones	DPP4 inhibitors	Thiazolidinediones
10-14	1	1	1	1
15-19	9	13	4	9
20-24	32	28	25	18
25-29	115	89	63	59
30-34	273	336	198	181
35-39	636	730	426	403
40-44	1378	1379	778	778
45-49	2261	2364	1277	1259
50-54	3613	3422	1911	1888
55-59	4151	3534	1974	2004
60-64	3627	2260	1494	1553
65-69	135	58	46	44
**Comparison**: DPP4 inhibitors vs Thiazolidinediones - **Outcome**: Kidney Disorders				
Age group	Unmatched		Matched	
	DPP4 inhibitors	Thiazolidinediones	DPP4 inhibitors	Thiazolidinediones
10-14	1	1	1	1
15-19	9	13	4	8
20-24	32	28	23	15
25-29	115	89	60	58
30-34	273	336	186	170
35-39	636	730	400	373
40-44	1378	1379	727	725
45-49	2261	2364	1222	1189
50-54	3613	3422	1770	1751
55-59	4151	3534	1772	1849
60-64	3627	2260	1344	1381
65-69	135	58	48	37
**Comparison**: DPP4 inhibitors vs Thiazolidinediones - **Outcome**: Eye Disorders				
Age group	Unmatched		Matched	
	DPP4 inhibitors	Thiazolidinediones	DPP4 inhibitors	Thiazolidinediones
10-14	1	1	1	1
15-19	9	13	5	10
20-24	32	28	25	16
25-29	115	89	63	55
30-34	273	336	189	171
35-39	636	730	411	396

© 2018 Vashisht R et al. *JAMA Network Open*.

40-44	1378	1379	727	741
45-49	2261	2364	1191	1143
50-54	3613	3422	1674	1679
55-59	4151	3534	1688	1725
60-64	3627	2260	1205	1251
65-69	135	58	41	32

© 2018 Vashisht R et al. *JAMA Network Open*.

eTable 8. Age Information Before and After Matching for Each Drug Comparison Based on the Data From Columbia University

**Comparison**: Sulfonylureas vs DPP4 inhibitors - **Outcome**: HbA1c				
Age group	Unmatched		Matched	
	Sulfonylureas	DPP4 inhibitors	Sulfonylureas	DPP4 inhibitors
25-29	24	2	2	2
30-34	26	4	1	4
35-39	49	8	1	3
40-44	121	3	3	3
45-49	175	20	14	13
50-54	280	32	21	23
55-59	304	46	30	28
60-64	336	52	41	26
65-69	312	49	28	26
70-74	275	47	17	31
75-79	168	30	16	17
80-84	116	28	17	11
85-89	54	24	13	14
90-94	15	7	1	3
95-99	2	1	NA	1
**Comparison**: Sulfonylureas vs DPP4 inhibitors - **Outcome**: Myocardial Infarction				
Age group	Unmatched		Matched	
	Sulfonylureas	DPP4 inhibitors	Sulfonylureas	DPP4 inhibitors
25-29	24	2	1	2
30-34	26	4	2	4
35-39	49	8	4	8
40-44	121	3	6	3
45-49	175	20	16	20
50-54	280	32	32	29
55-59	304	46	43	43
60-64	336	52	51	39
65-69	312	49	52	42
70-74	275	47	35	40
75-79	168	30	30	24
80-84	116	28	19	23
85-89	54	24	12	20
90-94	15	7	1	6
95-99	2	1	NA	1
**Comparison**: Sulfonylureas vs DPP4 inhibitors - **Outcome**: Kidney Disorders				
Age group	Unmatched		Matched	
	Sulfonylureas	DPP4 inhibitors	Sulfonylureas	DPP4 inhibitors
25-29	24	2	1	2
30-34	26	4	NA	3
35-39	49	8	2	8
40-44	121	3	7	3

© 2018 Vashisht R et al. *JAMA Network Open*.

45-49	175	20	17	19
50-54	280	32	31	26
55-59	304	46	37	38
60-64	336	52	45	38
65-69	312	49	49	42
70-74	275	47	34	40
75-79	168	30	21	20
80-84	116	28	21	21
85-89	54	24	14	18
90-94	15	7	2	2
95-99	2	1	NA	1
**Comparison**: Sulfonylureas vs DPP4 inhibitors - **Outcome**: Eye Disorders				
Age group	Unmatched		Matched	
	Sulfonylureas	DPP4 inhibitors	Sulfonylureas	DPP4 inhibitors
25-29	24	2	3	2
30-34	26	4	1	4
35-39	49	8	3	8
40-44	121	3	3	3
45-49	175	20	16	17
50-54	280	32	31	27
55-59	304	46	36	41
60-64	336	52	39	37
65-69	312	49	53	37
70-74	275	47	33	34
75-79	168	30	23	19
80-84	116	28	15	19
85-89	54	24	12	15
90-94	15	7	1	5
95-99	2	1	NA	1
**Comparison**: Sulfonylureas vs Thiazolidinediones - **Outcome**: HbA1c				
Age group	Unmatched		Matched	
	Sulfonylureas	Thiazolidinediones	Sulfonylureas	Thiazolidinediones
20-24	7	1	NA	1
25-29	23	NA	3	NA
30-34	27	3	4	1
35-39	47	9	7	6
40-44	120	17	15	6
45-49	166	39	23	28
50-54	267	52	36	37
55-59	309	53	33	29
60-64	313	59	48	42
65-69	305	64	36	35
70-74	254	52	29	34
75-79	156	40	13	26
80-84	107	22	12	15

© 2018 Vashisht R et al. *JAMA Network Open*.

85-89	54	11	4	7
90-94	16	3	5	2
95-99	2	NA	1	NA

**Comparison**: Sulfonylureas vs Thiazolidinediones - **Outcome**: Myocardial Infarction				
Age group	Unmatched		Matched	
	Sulfonylureas	Thiazolidinediones	Sulfonylureas	Thiazolidinediones
20-24	7	1	1	1
25-29	23	NA	3	NA
30-34	27	3	3	3
35-39	47	9	8	9
40-44	120	17	17	17
45-49	166	39	26	38
50-54	267	52	37	49
55-59	309	53	62	52
60-64	313	59	60	57
65-69	305	64	61	58
70-74	254	52	56	46
75-79	156	40	25	37
80-84	107	22	23	20
85-89	54	11	13	10
90-94	16	3	5	3
95-99	2	NA	NA	NA

**Comparison**: Sulfonylureas vs Thiazolidinediones - **Outcome**: Kidney Disorders				
Age group	Unmatched		Matched	
	Sulfonylureas	Thiazolidinediones	Sulfonylureas	Thiazolidinediones
20-24	7	1	1	1
25-29	23	NA	4	NA
30-34	27	3	4	2
35-39	47	9	6	9
40-44	120	17	17	16
45-49	166	39	28	37
50-54	267	52	29	40
55-59	309	53	64	47
60-64	313	59	52	57
65-69	305	64	60	60
70-74	254	52	51	45
75-79	156	40	25	31
80-84	107	22	19	16
85-89	54	11	9	9
90-94	16	3	3	2
95-99	2	NA	NA	NA

**Comparison**: Sulfonylureas vs Thiazolidinediones - **Outcome**: Eye Disorders				
Age group	Unmatched		Matched	
	Sulfonylureas	Thiazolidinediones	Sulfonylureas	Thiazolidinediones
20-24	7	1		

© 2018 Vashisht R et al. *JAMA Network Open*.

25-29	23	NA	1	1
30-34	27	3	5	NA
35-39	47	9	5	3
40-44	120	17	7	8
45-49	166	39	16	14
50-54	267	52	23	35
55-59	309	53	38	44
60-64	313	59	48	43
65-69	305	64	49	43
70-74	254	52	46	49
75-79	156	40	49	34
80-84	107	22	15	28
85-89	54	11	11	16
90-94	16	3	11	6
95-99	2	NA	NA	NA
**Comparison**: DPP4 inhibitors vs Thiazolidinediones - **Outcome**: HbA1c				
Age group	Unmatched		Matched	
	DPP4 inhibitors	Thiazolidinediones	DPP4 inhibitors	Thiazolidinediones
20-24	NA	2	NA	2
25-29	3	2	1	NA
30-34	4	4	3	NA
35-39	12	12	2	2
40-44	11	27	3	5
45-49	32	59	12	12
50-54	59	87	20	21
55-59	87	98	30	23
60-64	79	106	24	29
65-69	73	100	23	23
70-74	60	82	21	21
75-79	37	59	13	12
80-84	34	35	10	18
85-89	28	15	8	6
90-94	8	4	4	1
95-99	1	NA	1	NA
**Comparison**: DPP4 inhibitors vs Thiazolidinediones - **Outcome**: Myocardial Infarction				
Age group	Unmatched		Matched	
	DPP4 inhibitors	Thiazolidinediones	DPP4 inhibitors	Thiazolidinediones
20-24	NA	2	NA	2
25-29	3	2	1	1
30-34	4	4	3	1
35-39	12	12	6	1
40-44	11	27	2	12
45-49	32	59	17	19

© 2018 Vashisht R et al. *JAMA Network Open*.

50-54	59	87	32	29
55-59	87	98	38	34
60-64	79	106	27	37
65-69	73	100	33	33
70-74	60	82	29	23
75-79	37	59	17	20
80-84	34	35	16	18
85-89	28	15	10	4
90-94	8	4	5	3
95-99	1	NA	1	NA

Comparison: DPP4 inhibitors vs Thiazolidinediones - **Outcome**: Kidney Disorders

Age group	Unmatched		Matched	
	DPP4 inhibitors	Thiazolidinediones	DPP4 inhibitors	Thiazolidinediones
20-24	NA	2	NA	2
25-29	3	2	2	1
30-34	4	4	1	1
35-39	12	12	6	1
40-44	11	27	2	11
45-49	32	59	17	15
50-54	59	87	28	26
55-59	87	98	36	31
60-64	79	106	27	34
65-69	73	100	35	35
70-74	60	82	25	23
75-79	37	59	15	19
80-84	34	35	14	16
85-89	28	15	9	3
90-94	8	4	3	3
95-99	1	NA	1	NA

Comparison: DPP4 inhibitors vs Thiazolidinediones - **Outcome**: Eye Disorders

Age group	Unmatched		Matched	
	DPP4 inhibitors	Thiazolidinediones	DPP4 inhibitors	Thiazolidinediones
20-24	NA	2	NA	2
25-29	3	2	1	1
30-34	4	4	3	NA
35-39	12	12	7	2
40-44	11	27	2	11
45-49	32	59	16	12
50-54	59	87	29	26
55-59	87	98	29	31
60-64	79	106	30	29
65-69	73	100	28	30
70-74	60	82	21	18

© 2018 Vashisht R et al. *JAMA Network Open*.

75-79	37	59	12	21
80-84	34	35	14	16
85-89	28	15	6	4
90-94	8	4	5	1
95-99	1	NA	1	NA

© 2018 Vashisht R et al. *JAMA Network Open*.

eTable 9. Age Information Before and After Matching for Each Drug Comparison Based on the Data From IQVIA Disease Analyzer France

**Comparison**: Sulfonylureas vs DPP4 inhibitors - **Outcome**: HbA1c				
Age group	Unmatched		Matched	
	Sulfonylureas	DPP4 inhibitors	Sulfonylureas	DPP4 inhibitors
25-29	1	1	1	NA
30-34	5	8	4	3
35-39	16	17	11	5
40-44	44	64	27	32
45-49	86	101	44	45
50-54	159	168	72	69
55-59	220	272	124	116
60-64	300	326	151	143
65-69	279	269	130	110
70-74	239	210	95	106
75-79	152	160	68	82
80-84	123	82	30	44
85-89	25	16	13	13
90-94	3	5	1	3

**Comparison**: Sulfonylureas vs DPP4 inhibitors - **Outcome**: Myocardial Infarction				
Age group	Unmatched		Matched	
	Sulfonylureas	DPP4 inhibitors	Sulfonylureas	DPP4 inhibitors
25-29	1	1	1	NA
30-34	5	8	4	4
35-39	16	17	13	9
40-44	44	64	36	38
45-49	86	101	56	66
50-54	159	168	93	95
55-59	220	272	168	159
60-64	300	326	211	185
65-69	279	269	179	177
70-74	239	210	140	151
75-79	152	160	105	115
80-84	123	82	46	61
85-89	25	16	21	11
90-94	3	5	2	4

**Comparison**: Sulfonylureas vs DPP4 inhibitors - **Outcome**: Kidney Disorders				
Age group	Unmatched		Matched	
	Sulfonylureas	DPP4 inhibitors	Sulfonylureas	DPP4 inhibitors
25-29	1	1	1	NA
30-34	5	8	4	4
35-39	16	17	13	9
40-44	44	64	36	39
45-49	86	101	55	62
50-54	159	168	92	92

© 2018 Vashisht R et al. *JAMA Network Open*.

55-59	220	272	162	155
60-64	300	326	205	182
65-69	279	269	179	173
70-74	239	210	134	148
75-79	152	160	102	109
80-84	123	82	44	61
85-89	25	16	21	12
90-94	3	5	2	4
**Comparison**: Sulfonylureas vs DPP4 inhibitors - **Outcome**: Eye Disorders				
Age group	Unmatched		Matched	
	Sulfonylureas	DPP4 inhibitors	Sulfonylureas	DPP4 inhibitors
25-29	1	1	1	NA
30-34	5	8	4	4
35-39	16	17	13	8
40-44	44	64	36	38
45-49	86	101	57	67
50-54	159	168	94	98
55-59	220	272	168	160
60-64	300	326	211	182
65-69	279	269	180	177
70-74	239	210	137	151
75-79	152	160	107	116
80-84	123	82	46	60
85-89	25	16	20	12
90-94	3	5	3	4
**Comparison**: Sulfonylureas vs Thiazolidinediones - **Outcome**: HbA1c				
Age group	Unmatched		Matched	
	Sulfonylureas	Thiazolidinediones	Sulfonylureas	Thiazolidinediones
20-24	NA	1	NA	1
25-29	1	3	1	2
30-34	6	3	1	3
35-39	20	9	4	7
40-44	46	18	10	11
45-49	97	52	27	27
50-54	166	108	68	58
55-59	242	143	91	89
60-64	315	162	105	94
65-69	310	125	68	75
70-74	243	117	70	72
75-79	163	61	34	32
80-84	131	23	9	18
85-89	26	4	2	1
90-94	5	1	NA	NA
**Comparison**: Sulfonylureas vs Thiazolidinediones - **Outcome**: Myocardial Infarction				
Age	Unmatched		Matched	

© 2018 Vashisht R et al. *JAMA Network Open*.

group	Sulfonylureas	Thiazolidinediones	Sulfonylureas	Thiazolidinediones
20-24	NA	1	NA	1
25-29	1	3	1	3
30-34	6	3	1	3
35-39	20	9	3	7
40-44	46	18	17	13
45-49	97	52	34	36
50-54	166	108	78	62
55-59	242	143	103	106
60-64	315	162	125	115
65-69	310	125	80	95
70-74	243	117	81	81
75-79	163	61	46	42
80-84	131	23	14	22
85-89	26	4	4	1
90-94	5	1	1	1

Comparison: Sulfonylureas vs Thiazolidinediones - **Outcome**: Kidney Disorders

Age group	Unmatched		Matched	
	Sulfonylureas	Thiazolidinediones	Sulfonylureas	Thiazolidinediones
20-24	NA	1	NA	1
25-29	1	3	1	3
30-34	6	3	2	3
35-39	20	9	1	6
40-44	46	18	17	13
45-49	97	52	34	35
50-54	166	108	78	61
55-59	242	143	100	104
60-64	315	162	123	113
65-69	310	125	78	89
70-74	243	117	77	81
75-79	163	61	44	42
80-84	131	23	13	21
85-89	26	4	5	1
90-94	5	1	1	1

Comparison: Sulfonylureas vs Thiazolidinediones - **Outcome**: Eye Disorders

Age group	Unmatched		Matched	
	Sulfonylureas	Thiazolidinediones	Sulfonylureas	Thiazolidinediones
20-24	NA	1	NA	1
25-29	1	3	1	3
30-34	6	3	1	3
35-39	20	9	2	7
40-44	46	18	16	13
45-49	97	52	33	36
50-54	166	108	77	61
55-59	242	143	103	108

© 2018 Vashisht R et al. *JAMA Network Open*.

60-64	315	162	126	117
65-69	310	125	79	94
70-74	243	117	82	80
75-79	163	61	48	41
80-84	131	23	13	20
85-89	26	4	4	1
90-94	5	1	1	1
**Comparison**: DPP4 inhibitors vs Thiazolidinediones - **Outcome**: HbA1c				
Age group	Unmatched		Matched	
	DPP4 inhibitors	Thiazolidinediones	DPP4 inhibitors	Thiazolidinediones
20-24	NA	1	NA	1
25-29	1	3	NA	2
30-34	9	3	2	3
35-39	22	8	4	6
40-44	63	20	13	12
45-49	113	56	21	22
50-54	185	110	47	45
55-59	299	136	69	75
60-64	344	155	65	70
65-69	282	122	58	48
70-74	216	129	60	54
75-79	177	66	28	33
80-84	91	24	10	16
85-89	19	6	9	1
90-94	7	1	2	NA
**Comparison**: DPP4 inhibitors vs Thiazolidinediones - **Outcome**: Myocardial Infarction				
Age group	Unmatched		Matched	
	DPP4 inhibitors	Thiazolidinediones	DPP4 inhibitors	Thiazolidinediones
20-24	NA	1	NA	1
25-29	1	3	NA	3
30-34	9	3	2	3
35-39	22	8	6	7
40-44	63	20	17	15
45-49	113	56	30	29
50-54	185	110	65	57
55-59	299	136	88	94
60-64	344	155	88	97
65-69	282	122	76	74
70-74	216	129	71	63
75-79	177	66	39	39
80-84	91	24	15	21
85-89	19	6	7	2
90-94	7	1	2	1

© 2018 Vashisht R et al. *JAMA Network Open*.

Comparison: DPP4 inhibitors vs Thiazolidinediones - Outcome: Kidney Disorders				
Age group	Unmatched		Matched	
	DPP4 inhibitors	Thiazolidinediones	DPP4 inhibitors	Thiazolidinediones
20-24	NA	1	NA	1
25-29	1	3	NA	3
30-34	9	3	2	3
35-39	22	8	5	7
40-44	63	20	18	15
45-49	113	56	30	29
50-54	185	110	64	56
55-59	299	136	86	93
60-64	344	155	86	93
65-69	282	122	77	73
70-74	216	129	71	62
75-79	177	66	34	39
80-84	91	24	16	20
85-89	19	6	7	2
90-94	7	1	1	1

Comparison: DPP4 inhibitors vs Thiazolidinediones - Outcome: Eye Disorders				
Age group	Unmatched		Matched	
	DPP4 inhibitors	Thiazolidinediones	DPP4 inhibitors	Thiazolidinediones
20-24	NA	1	NA	1
25-29	1	3	NA	3
30-34	9	3	2	3
35-39	22	8	6	7
40-44	63	20	16	15
45-49	113	56	30	28
50-54	185	110	65	57
55-59	299	136	87	95
60-64	344	155	88	97
65-69	282	122	77	75
70-74	216	129	73	63
75-79	177	66	37	37
80-84	91	24	15	21
85-89	19	6	8	2
90-94	7	1	1	1

© 2018 Vashisht R et al. *JAMA Network Open*.

eTable 10. Age Information Before and After Matching for Each Drug Comparison Based on the Data From Truven MarketScan MDCR

**Comparison**: Sulfonylureas vs DPP4 inhibitors - **Outcome**: HbA1c				
Age group	Unmatched		Matched	
	Sulfonylureas	DPP4 inhibitors	Sulfonylureas	DPP4 inhibitors
45-49	4	1	1	1
50-54	19	3	3	2
55-59	41	20	22	18
60-64	76	34	37	32
65-69	3369	948	796	775
70-74	1659	532	388	414
75-79	944	288	211	218
80-84	527	182	146	142
85-89	180	59	46	48
90-94	43	13	9	10
95-99	4	1	2	1
**Comparison**: Sulfonylureas vs DPP4 inhibitors - **Outcome**: Myocardial Infarction				
Age group	Unmatched		Matched	
	Sulfonylureas	DPP4 inhibitors	Sulfonylureas	DPP4 inhibitors
45-49	4	1	NA	1
50-54	19	3	8	3
55-59	41	20	22	18
60-64	76	34	37	31
65-69	3369	948	945	933
70-74	1659	532	463	519
75-79	944	288	269	268
80-84	527	182	183	171
85-89	180	59	70	51
90-94	43	13	10	12
95-99	4	1	1	1
**Comparison**: Sulfonylureas vs DPP4 inhibitors - **Outcome**: Kidney Disorders				
Age group	Unmatched		Matched	
	Sulfonylureas	DPP4 inhibitors	Sulfonylureas	DPP4 inhibitors
45-49	4	1	NA	1
50-54	19	3	3	3
55-59	41	20	13	14
60-64	76	34	31	28
65-69	3369	948	784	786
70-74	1659	532	369	389
75-79	944	288	188	191
80-84	527	182	125	106
85-89	180	59	36	29
90-94	43	13	4	7
95-99	4	1	1	NA
**Comparison**: Sulfonylureas vs DPP4 inhibitors - **Outcome**: Eye Disorders				

© 2018 Vashisht R et al. *JAMA Network Open*.

Age group	Unmatched		Matched	
	Sulfonylureas	DPP4 inhibitors	Sulfonylureas	DPP4 inhibitors
45-49	4	1	1	1
50-54	19	3	7	3
55-59	41	20	18	15
60-64	76	34	26	27
65-69	3369	948	642	676
70-74	1659	532	253	266
75-79	944	288	141	130
80-84	527	182	92	82
85-89	180	59	39	23
90-94	43	13	6	4
95-99	4	1	2	NA

Comparison: Sulfonylureas vs Thiazolidinediones - **Outcome**: HbA1c

Age group	Unmatched		Matched	
	Sulfonylureas	Thiazolidinediones	Sulfonylureas	Thiazolidinediones
40-44	NA	1	NA	1
45-49	2	2	NA	2
50-54	20	7	3	7
55-59	42	23	15	19
60-64	73	21	20	19
65-69	3324	757	670	663
70-74	1630	424	377	370
75-79	933	250	227	219
80-84	522	108	97	93
85-89	188	42	25	37
90-94	41	12	6	10

Comparison: Sulfonylureas vs Thiazolidinediones - **Outcome**: Myocardial Infarction

Age group	Unmatched		Matched	
	Sulfonylureas	Thiazolidinediones	Sulfonylureas	Thiazolidinediones
40-44	NA	1	NA	1
45-49	2	2	NA	2
50-54	20	7	3	7
55-59	42	23	15	20
60-64	73	21	22	19
65-69	3324	757	725	743
70-74	1630	424	405	400
75-79	933	250	253	236
80-84	522	108	118	99
85-89	188	42	30	39
90-94	41	12	7	12

Comparison: Sulfonylureas vs Thiazolidinediones - **Outcome**: Kidney Disorders

Age group	Unmatched		Matched	
	Sulfonylureas	Thiazolidinediones	Sulfonylureas	Thiazolidinediones
40-44	NA	1	NA	1

© 2018 Vashisht R et al. *JAMA Network Open*.

45-49	2	2	NA	1
50-54	20	7	4	7
55-59	42	23	18	18
60-64	73	21	19	15
65-69	3324	757	609	623
70-74	1630	424	330	326
75-79	933	250	189	178
80-84	522	108	76	71
85-89	188	42	23	24
90-94	41	12	4	8

Comparison: Sulfonylureas vs Thiazolidinediones - **Outcome**: Eye Disorders

Age group	Unmatched		Matched	
	Sulfonylureas	Thiazolidinediones	Sulfonylureas	Thiazolidinediones
40-44	NA	1	NA	1
45-49	2	2	NA	2
50-54	20	7	4	5
55-59	42	23	12	20
60-64	73	21	14	14
65-69	3324	757	565	566
70-74	1630	424	267	237
75-79	933	250	130	139
80-84	522	108	54	53
85-89	188	42	13	20
90-94	41	12	4	6

Comparison: DPP4 inhibitors vs Thiazolidinediones - **Outcome**: HbA1c

Age group	Unmatched		Matched	
	DPP4 inhibitors	Thiazolidinediones	DPP4 inhibitors	Thiazolidinediones
45-49	1	4	NA	1
50-54	6	10	1	5
55-59	23	25	9	13
60-64	34	21	19	15
65-69	1157	1021	490	457
70-74	629	536	186	217
75-79	333	309	129	125
80-84	210	147	83	81
85-89	74	47	22	21
90-94	13	13	3	7

Comparison: DPP4 inhibitors vs Thiazolidinediones - **Outcome**: Myocardial Infarction

Age group	Unmatched		Matched	
	DPP4 inhibitors	Thiazolidinediones	DPP4 inhibitors	Thiazolidinediones
45-49	1	4	NA	1
50-54	6	10	1	5

© 2018 Vashisht R et al. *JAMA Network Open*.

55-59	23	25	9	16
60-64	34	21	17	15
65-69	1157	1021	553	529
70-74	629	536	217	245
75-79	333	309	151	147
80-84	210	147	93	79
85-89	74	47	28	28
90-94	13	13	3	7

Comparison: DPP4 inhibitors vs Thiazolidinediones - **Outcome**: Kidney Disorders

Age group	Unmatched		Matched	
	DPP4 inhibitors	Thiazolidinediones	DPP4 inhibitors	Thiazolidinediones
45-49	1	4	NA	1
50-54	6	10	1	5
55-59	23	25	8	13
60-64	34	21	17	13
65-69	1157	1021	490	455
70-74	629	536	175	205
75-79	333	309	112	116
80-84	210	147	65	57
85-89	74	47	20	18
90-94	13	13	NA	5

Comparison: DPP4 inhibitors vs Thiazolidinediones - **Outcome**: Eye Disorders

Age group	Unmatched		Matched	
	DPP4 inhibitors	Thiazolidinediones	DPP4 inhibitors	Thiazolidinediones
45-49	1	4	NA	1
50-54	6	10	NA	3
55-59	23	25	6	14
60-64	34	21	13	11
65-69	1157	1021	437	404
70-74	629	536	135	149
75-79	333	309	82	85
80-84	210	147	48	45
85-89	74	47	10	17
90-94	13	13	1	3

© 2018 Vashisht R et al. *JAMA Network Open*.

eTable 11. Age Information Before and After Matching for Each Drug Comparison Based on the Data From Mount Sinai

**Comparison**: Sulfonylureas vs DPP4 inhibitors - **Outcome**: HbA1c				
Age group	Unmatched		Matched	
	Sulfonylureas	DPP4 inhibitors	Sulfonylureas	DPP4 inhibitors
15-19	1	2	1	1
20-24	1	2	NA	2
25-29	6	5	1	3
30-34	22	6	7	5
35-39	49	14	10	13
40-44	85	37	29	30
45-49	144	61	54	45
50-54	240	125	85	96
55-59	317	179	132	140
60-64	297	186	148	145
65-69	369	172	147	135
70-74	261	144	109	115
75-79	195	107	82	83
80-84	116	65	51	47
85-89	57	16	16	14
90-94	15	6	8	6
**Comparison**: Sulfonylureas vs DPP4 inhibitors - **Outcome**: Myocardial Infarction				
Age group	Unmatched		Matched	
	Sulfonylureas	DPP4 inhibitors	Sulfonylureas	DPP4 inhibitors
15-19	1	2	NA	2
20-24	1	2	1	2
25-29	6	5	3	4
30-34	22	6	13	6
35-39	49	14	16	14
40-44	85	37	31	34
45-49	144	61	66	57
50-54	240	125	108	118
55-59	317	179	158	162
60-64	297	186	173	171
65-69	369	172	164	161
70-74	261	144	129	131
75-79	195	107	90	97
80-84	116	65	62	61
85-89	57	16	19	15
90-94	15	6	8	6
**Comparison**: Sulfonylureas vs DPP4 inhibitors - **Outcome**: Kidney Disorders				
Age group	Unmatched		Matched	
	Sulfonylureas	DPP4 inhibitors	Sulfonylureas	DPP4 inhibitors
15-19	1	2	NA	1
20-24	1	2	1	2

© 2018 Vashisht R et al. *JAMA Network Open*.

25-29	6	5	1	4
30-34	22	6	10	6
35-39	49	14	19	14
40-44	85	37	30	33
45-49	144	61	63	55
50-54	240	125	101	116
55-59	317	179	151	162
60-64	297	186	165	167
65-69	369	172	166	153
70-74	261	144	132	130
75-79	195	107	90	94
80-84	116	65	57	56
85-89	57	16	19	14
90-94	15	6	8	6
**Comparison**: Sulfonylureas vs DPP4 inhibitors - **Outcome**: Eye Disorders				
Age group	Unmatched		Matched	
	Sulfonylureas	DPP4 inhibitors	Sulfonylureas	DPP4 inhibitors
15-19	1	2	NA	2
20-24	1	2	1	2
25-29	6	5	2	4
30-34	22	6	11	6
35-39	49	14	16	14
40-44	85	37	28	34
45-49	144	61	63	55
50-54	240	125	106	116
55-59	317	179	153	161
60-64	297	186	163	165
65-69	369	172	161	150
70-74	261	144	127	127
75-79	195	107	88	95
80-84	116	65	60	55
85-89	57	16	18	15
90-94	15	6	9	5
**Comparison**: Sulfonylureas vs Thiazolidinediones - **Outcome**: HbA1c				
Age group	Unmatched		Matched	
	Sulfonylureas	Thiazolidinediones	Sulfonylureas	Thiazolidinediones
25-29	4	1	NA	1
30-34	24	4	2	2
35-39	47	11	10	9
40-44	96	24	17	11
45-49	150	45	25	40
50-54	261	70	49	55
55-59	334	72	48	57
60-64	348	80	73	65
65-69	403	84	77	68

© 2018 Vashisht R et al. *JAMA Network Open*.

70-74	306	48	38	39
75-79	228	48	33	38
80-84	132	33	28	25
85-89	63	6	13	5
90-94	14	3	2	2
95-99	3	NA	2	NA
**Comparison**: Sulfonylureas vs Thiazolidinediones - **Outcome**: Myocardial Infarction				
Age group	Unmatched		Matched	
	Sulfonylureas	Thiazolidinediones	Sulfonylureas	Thiazolidinediones
25-29	4	1	1	1
30-34	24	4	4	4
35-39	47	11	9	11
40-44	96	24	24	24
45-49	150	45	33	44
50-54	261	70	58	66
55-59	334	72	59	72
60-64	348	80	88	78
65-69	403	84	93	80
70-74	306	48	52	48
75-79	228	48	48	47
80-84	132	33	30	33
85-89	63	6	15	6
90-94	14	3	2	3
95-99	3	NA	1	NA
**Comparison**: Sulfonylureas vs Thiazolidinediones - **Outcome**: Kidney Disorders				
Age group	Unmatched		Matched	
	Sulfonylureas	Thiazolidinediones	Sulfonylureas	Thiazolidinediones
25-29	4	1	NA	1
30-34	24	4	4	4
35-39	47	11	9	11
40-44	96	24	26	24
45-49	150	45	28	43
50-54	261	70	54	65
55-59	334	72	56	68
60-64	348	80	86	75
65-69	403	84	100	79
70-74	306	48	51	46
75-79	228	48	45	44
80-84	132	33	25	32
85-89	63	6	13	6
90-94	14	3	2	3
95-99	3	NA	2	NA
**Comparison**: Sulfonylureas vs Thiazolidinediones - **Outcome**: Eye Disorders				
Age group	Unmatched		Matched	
	Sulfonylureas	Thiazolidinediones	Sulfonylureas	Thiazolidinediones

© 2018 Vashisht R et al. *JAMA Network Open*.

25-29	4	1	1	1
30-34	24	4	2	4
35-39	47	11	10	10
40-44	96	24	22	21
45-49	150	45	29	38
50-54	261	70	56	65
55-59	334	72	53	61
60-64	348	80	80	75
65-69	403	84	81	72
70-74	306	48	45	43
75-79	228	48	47	44
80-84	132	33	29	32
85-89	63	6	14	4
90-94	14	3	1	2
95-99	3	NA	2	NA
**Comparison**: DPP4 inhibitors vs Thiazolidinediones - **Outcome**: HbA1c				
Age group	Unmatched		Matched	
	DPP4 inhibitors	Thiazolidinediones	DPP4 inhibitors	Thiazolidinediones
25-29	5	3	NA	1
30-34	10	7	2	2
35-39	16	14	8	7
40-44	56	34	18	17
45-49	82	61	33	34
50-54	172	97	55	65
55-59	253	129	70	72
60-64	275	109	83	74
65-69	245	129	73	76
70-74	213	73	50	51
75-79	154	60	48	41
80-84	91	43	22	26
85-89	26	10	11	6
90-94	7	5	2	3
**Comparison**: DPP4 inhibitors vs Thiazolidinediones - **Outcome**: Myocardial Infarction				
Age group	Unmatched		Matched	
	DPP4 inhibitors	Thiazolidinediones	DPP4 inhibitors	Thiazolidinediones
25-29	5	3	1	3
30-34	10	7	2	4
35-39	16	14	8	10
40-44	56	34	19	22
45-49	82	61	38	40
50-54	172	97	72	72
55-59	253	129	79	84

© 2018 Vashisht R et al. *JAMA Network Open*.

60-64	275	109	95	85
65-69	245	129	81	89
70-74	213	73	59	57
75-79	154	60	59	47
80-84	91	43	28	29
85-89	26	10	11	8
90-94	7	5	2	4

Comparison: DPP4 inhibitors vs Thiazolidinediones - **Outcome**: Kidney Disorders

Age group	Unmatched		Matched	
	DPP4 inhibitors	Thiazolidinediones	DPP4 inhibitors	Thiazolidinediones
25-29	5	3	1	3
30-34	10	7	2	4
35-39	16	14	7	10
40-44	56	34	19	22
45-49	82	61	36	40
50-54	172	97	71	72
55-59	253	129	79	78
60-64	275	109	94	81
65-69	245	129	81	88
70-74	213	73	59	54
75-79	154	60	52	46
80-84	91	43	26	29
85-89	26	10	9	7
90-94	7	5	2	4

Comparison: DPP4 inhibitors vs Thiazolidinediones - **Outcome**: Eye Disorders

Age group	Unmatched		Matched	
	DPP4 inhibitors	Thiazolidinediones	DPP4 inhibitors	Thiazolidinediones
25-29	5	3	1	2
30-34	10	7	2	4
35-39	16	14	8	10
40-44	56	34	17	19
45-49	82	61	39	37
50-54	172	97	67	73
55-59	253	129	76	79
60-64	275	109	93	82
65-69	245	129	76	84
70-74	213	73	58	53
75-79	154	60	53	47
80-84	91	43	26	29
85-89	26	10	12	7
90-94	7	5	1	3

© 2018 Vashisht R et al. *JAMA Network Open*.

eTable 12. Age Information Before and After Matching for Each Drug Comparison Based on the Data From Optum Clinformatics Data Mart

**Comparison**: Sulfonylureas vs DPP4 inhibitors - **Outcome**: HbA1c				
Age group	Unmatched		Matched	
	Sulfonylureas	DPP4 inhibitors	Sulfonylureas	DPP4 inhibitors
5-9	NA	1	NA	NA
10-14	3	3	1	1
15-19	35	10	6	9
20-24	145	39	31	34
25-29	632	162	120	134
30-34	1713	441	333	332
35-39	3265	1131	847	851
40-44	5634	2144	1570	1535
45-49	8604	3367	2424	2385
50-54	11197	4641	3278	3195
55-59	12182	5291	3479	3489
60-64	10776	4878	3235	3098
65-69	15806	5704	3990	4138
70-74	9649	4053	2710	2765
75-79	6003	2353	1511	1574
80-84	2815	1433	957	954
85-89	692	469	285	283
**Comparison**: Sulfonylureas vs DPP4 inhibitors - **Outcome**: Myocardial Infarction				
Age group	Unmatched		Matched	
	Sulfonylureas	DPP4 inhibitors	Sulfonylureas	DPP4 inhibitors
5-9	NA	1	NA	1
10-14	3	3	1	3
15-19	35	10	8	10
20-24	145	39	38	39
25-29	632	162	153	160
30-34	1713	441	411	437
35-39	3265	1131	1063	1103
40-44	5634	2144	2026	2077
45-49	8604	3367	3220	3255
50-54	11197	4641	4380	4421
55-59	12182	5291	4899	4975
60-64	10776	4878	4702	4553
65-69	15806	5704	5484	5489
70-74	9649	4053	3909	3842
75-79	6003	2353	2189	2197
80-84	2815	1433	1381	1318
85-89	692	469	439	423
**Comparison**: Sulfonylureas vs DPP4 inhibitors - **Outcome**: Kidney Disorders				
Age group	Unmatched		Matched	
	Sulfonylureas	DPP4 inhibitors	Sulfonylureas	DPP4 inhibitors

© 2018 Vashisht R et al. *JAMA Network Open*.

5-9	NA	1	NA	1
10-14	3	3	1	3
15-19	35	10	8	9
20-24	145	39	36	37
25-29	632	162	139	150
30-34	1713	441	393	408
35-39	3265	1131	998	1016
40-44	5634	2144	1870	1883
45-49	8604	3367	2858	2934
50-54	11197	4641	3884	3894
55-59	12182	5291	4305	4365
60-64	10776	4878	4021	3963
65-69	15806	5704	4608	4574
70-74	9649	4053	3024	3067
75-79	6003	2353	1648	1602
80-84	2815	1433	974	902
85-89	692	469	293	252
**Comparison**: Sulfonylureas vs DPP4 inhibitors - **Outcome**: Eye Disorders				
Age group	Unmatched		Matched	
	Sulfonylureas	DPP4 inhibitors	Sulfonylureas	DPP4 inhibitors
5-9	NA	1	NA	1
10-14	3	3	1	3
15-19	35	10	10	10
20-24	145	39	31	39
25-29	632	162	146	153
30-34	1713	441	368	404
35-39	3265	1131	1005	1045
40-44	5634	2144	1867	1904
45-49	8604	3367	2848	2890
50-54	11197	4641	3783	3770
55-59	12182	5291	4014	4035
60-64	10776	4878	3587	3399
65-69	15806	5704	3880	3924
70-74	9649	4053	2440	2448
75-79	6003	2353	1351	1367
80-84	2815	1433	836	788
85-89	692	469	293	280
**Comparison**: Sulfonylureas vs Thiazolidinediones - **Outcome**: HbA1c				
Age group	Unmatched		Matched	
	Sulfonylureas	Thiazolidinediones	Sulfonylureas	Thiazolidinediones
5-9	NA	1	NA	1
10-14	2	1	NA	1
15-19	35	12	15	10
20-24	141	37	42	30
25-29	610	246	195	214

© 2018 Vashisht R et al. *JAMA Network Open*.

30-34	1668	605	542	504
35-39	3191	1320	1133	1078
40-44	5505	2320	1906	1867
45-49	8432	3491	2827	2721
50-54	11135	4554	3591	3497
55-59	12119	4906	3758	3637
60-64	10845	3780	2795	2756
65-69	16398	3767	2706	2990
70-74	9882	2355	1673	1830
75-79	6044	1554	1171	1212
80-84	2989	740	513	529
85-89	763	122	86	76
**Comparison**: Sulfonylureas vs Thiazolidinediones - **Outcome**: Myocardial Infarction				
Age group	Unmatched		Matched	
	Sulfonylureas	Thiazolidinediones	Sulfonylureas	Thiazolidinediones
5-9	NA	1	NA	1
10-14	2	1	1	1
15-19	35	12	16	12
20-24	141	37	52	37
25-29	610	246	224	242
30-34	1668	605	631	594
35-39	3191	1320	1346	1278
40-44	5505	2320	2267	2224
45-49	8432	3491	3402	3318
50-54	11135	4554	4374	4303
55-59	12119	4906	4717	4625
60-64	10845	3780	3704	3597
65-69	16398	3767	3396	3680
70-74	9882	2355	2185	2295
75-79	6044	1554	1448	1502
80-84	2989	740	670	721
85-89	763	122	116	119
**Comparison**: Sulfonylureas vs Thiazolidinediones - **Outcome**: Kidney Disorders				
Age group	Unmatched		Matched	
	Sulfonylureas	Thiazolidinediones	Sulfonylureas	Thiazolidinediones
5-9	NA	1	NA	1
10-14	2	1	1	1
15-19	35	12	14	11
20-24	141	37	52	35
25-29	610	246	205	236
30-34	1668	605	602	570
35-39	3191	1320	1267	1209
40-44	5505	2320	2164	2087
45-49	8432	3491	3199	3106
50-54	11135	4554	4059	4003

© 2018 Vashisht R et al. *JAMA Network Open*.

55-59	12119	4906	4307	4257
60-64	10845	3780	3299	3244
65-69	16398	3767	2984	3241
70-74	9882	2355	1807	1944
75-79	6044	1554	1211	1229
80-84	2989	740	512	534
85-89	763	122	92	67
**Comparison**: Sulfonylureas vs Thiazolidinediones - **Outcome**: Eye Disorders				
Age group	Unmatched		Matched	
	Sulfonylureas	Thiazolidinediones	Sulfonylureas	Thiazolidinediones
5-9	NA	1	NA	1
10-14	2	1	1	1
15-19	35	12	16	11
20-24	141	37	50	37
25-29	610	246	217	236
30-34	1668	605	597	568
35-39	3191	1320	1298	1214
40-44	5505	2320	2116	2089
45-49	8432	3491	3119	3046
50-54	11135	4554	3894	3786
55-59	12119	4906	4020	3911
60-64	10845	3780	2840	2844
65-69	16398	3767	2601	2797
70-74	9882	2355	1512	1625
75-79	6044	1554	968	1045
80-84	2989	740	445	490
85-89	763	122	79	72
**Comparison**: DPP4 inhibitors vs Thiazolidinediones - **Outcome**: HbA1c				
Age group	Unmatched		Matched	
	DPP4 inhibitors	Thiazolidinediones	DPP4 inhibitors	Thiazolidinediones
5-9	1	1	NA	NA
10-14	3	1	1	NA
15-19	11	14	8	6
20-24	46	53	32	25
25-29	196	303	118	123
30-34	529	748	308	304
35-39	1357	1641	688	707
40-44	2560	2904	1185	1242
45-49	4076	4388	1794	1719
50-54	5614	5612	2306	2274
55-59	6352	6002	2471	2419
60-64	5871	4653	2035	2067
65-69	7255	4837	2722	2738
70-74	4952	2913	1561	1622

© 2018 Vashisht R et al. *JAMA Network Open*.

75-79	2771	1922	909	924
80-84	1697	821	518	509
85-89	575	136	107	84

**Comparison**: DPP4 inhibitors vs Thiazolidinediones - **Outcome**: Myocardial Infarction				
Age group	Unmatched		Matched	
	DPP4 inhibitors	Thiazolidinediones	DPP4 inhibitors	Thiazolidinediones
5-9	1	1	1	NA
10-14	3	1	3	NA
15-19	11	14	10	7
20-24	46	53	34	33
25-29	196	303	142	146
30-34	529	748	369	366
35-39	1357	1641	873	857
40-44	2560	2904	1534	1499
45-49	4076	4388	2291	2215
50-54	5614	5612	2942	2852
55-59	6352	6002	3229	3133
60-64	5871	4653	2764	2774
65-69	7255	4837	3292	3452
70-74	4952	2913	2018	2116
75-79	2771	1922	1178	1222
80-84	1697	821	682	700
85-89	575	136	143	133

**Comparison**: DPP4 inhibitors vs Thiazolidinediones - **Outcome**: Kidney Disorders				
Age group	Unmatched		Matched	
	DPP4 inhibitors	Thiazolidinediones	DPP4 inhibitors	Thiazolidinediones
5-9	1	1	1	NA
10-14	3	1	3	NA
15-19	11	14	10	6
20-24	46	53	31	32
25-29	196	303	137	133
30-34	529	748	347	352
35-39	1357	1641	795	790
40-44	2560	2904	1401	1379
45-49	4076	4388	2111	2022
50-54	5614	5612	2632	2606
55-59	6352	6002	2911	2847
60-64	5871	4653	2452	2451
65-69	7255	4837	2922	3002
70-74	4952	2913	1669	1757
75-79	2771	1922	891	968
80-84	1697	821	515	502

© 2018 Vashisht R et al. *JAMA Network Open*.

85-89	575	136	99	80
**Comparison**: DPP4 inhibitors vs Thiazolidinediones - **Outcome**: Eye Disorders				
Age group	Unmatched		Matched	
	DPP4 inhibitors	Thiazolidinediones	DPP4 inhibitors	Thiazolidinediones
5-9	1	1	1	NA
10-14	3	1	3	NA
15-19	11	14	10	6
20-24	46	53	33	31
25-29	196	303	134	133
30-34	529	748	349	336
35-39	1357	1641	829	795
40-44	2560	2904	1398	1391
45-49	4076	4388	2084	2012
50-54	5614	5612	2560	2490
55-59	6352	6002	2704	2655
60-64	5871	4653	2115	2181
65-69	7255	4837	2511	2612
70-74	4952	2913	1396	1465
75-79	2771	1922	796	804
80-84	1697	821	439	472
85-89	575	136	108	87

© 2018 Vashisht R et al. *JAMA Network Open*.

eTable 13. Age Information Before and After Matching for Each Drug Comparison Based on the Data From Ajou University, South Korea

**Comparison**: Sulfonylureas vs DPP4 inhibitors - **Outcome**: HbA1c				
Age group	Unmatched		Matched	
	Sulfonylureas	DPP4 inhibitors	Sulfonylureas	DPP4 inhibitors
15-19	6	4	2	4
20-24	8	2	6	2
25-29	22	4	7	3
30-34	38	12	11	10
35-39	88	43	43	35
40-44	130	72	43	47
45-49	198	100	56	63
50-54	204	148	88	95
55-59	190	138	79	80
60-64	180	126	67	75
65-69	146	111	64	64
70-74	140	106	63	60
75-79	61	50	31	23
80-84	23	23	13	13
85-89	8	5	3	2
**Comparison**: Sulfonylureas vs DPP4 inhibitors - **Outcome**: Myocardial Infarction				
Age group	Unmatched		Matched	
	Sulfonylureas	DPP4 inhibitors	Sulfonylureas	DPP4 inhibitors
15-19	6	4	2	4
20-24	8	2	5	2
25-29	22	4	7	3
30-34	38	12	13	9
35-39	88	43	40	35
40-44	130	72	47	50
45-49	198	100	60	67
50-54	204	148	90	97
55-59	190	138	79	81
60-64	180	126	69	79
65-69	146	111	70	65
70-74	140	106	68	64
75-79	61	50	31	28
80-84	23	23	14	13
85-89	8	5	4	2
**Comparison**: Sulfonylureas vs DPP4 inhibitors - **Outcome**: Kidney Disorders				
Age group	Unmatched		Matched	
	Sulfonylureas	DPP4 inhibitors	Sulfonylureas	DPP4 inhibitors
15-19	6	4	2	4
20-24	8	2	5	2
25-29	22	4	7	3
30-34	38	12	10	10

© 2018 Vashisht R et al. *JAMA Network Open*.

35-39	88	43	40	33
40-44	130	72	48	51
45-49	198	100	61	67
50-54	204	148	90	97
55-59	190	138	78	81
60-64	180	126	67	77
65-69	146	111	68	65
70-74	140	106	72	62
75-79	61	50	29	27
80-84	23	23	14	13
85-89	8	5	3	2
**Comparison**: Sulfonylureas vs DPP4 inhibitors - **Outcome**: Eye Disorders				
Age group	Unmatched		Matched	
	Sulfonylureas	DPP4 inhibitors	Sulfonylureas	DPP4 inhibitors
15-19	6	4	2	4
20-24	8	2	5	2
25-29	22	4	7	3
30-34	38	12	12	10
35-39	88	43	40	35
40-44	130	72	46	51
45-49	198	100	63	65
50-54	204	148	87	98
55-59	190	138	80	81
60-64	180	126	69	77
65-69	146	111	70	66
70-74	140	106	71	63
75-79	61	50	30	28
80-84	23	23	14	14
85-89	8	5	3	2
**Comparison**: Sulfonylureas vs Thiazolidinediones - **Outcome**: HbA1c				
Age group	Unmatched		Matched	
	Sulfonylureas	Thiazolidinediones	Sulfonylureas	Thiazolidinediones
15-19	7	1	NA	1
20-24	10	2	NA	2
25-29	26	1	NA	1
30-34	53	3	4	3
35-39	97	9	6	8
40-44	154	22	15	20
45-49	236	24	26	21
50-54	255	32	26	30
55-59	232	28	27	23
60-64	212	32	24	27
65-69	176	24	24	20
70-74	171	14	10	12
75-79	72	6	8	5

© 2018 Vashisht R et al. *JAMA Network Open*.

80-84	24	2	3	2
85-89	8	NA	2	NA
90-94	1	NA	NA	NA

Comparison: Sulfonylureas vs Thiazolidinediones - **Outcome**: Myocardial Infarction

Age group	Unmatched		Matched	
	Sulfonylureas	Thiazolidinediones	Sulfonylureas	Thiazolidinediones
15-19	7	1	NA	1
20-24	10	2	NA	2
25-29	26	1	NA	1
30-34	53	3	3	3
35-39	97	9	8	8
40-44	154	22	14	21
45-49	236	24	28	22
50-54	255	32	26	31
55-59	232	28	27	23
60-64	212	32	25	29
65-69	176	24	23	22
70-74	171	14	11	12
75-79	72	6	10	4
80-84	24	2	4	2
85-89	8	NA	2	NA
90-94	1	NA	NA	NA

Comparison: Sulfonylureas vs Thiazolidinediones - **Outcome**: Kidney Disorders

Age group	Unmatched		Matched	
	Sulfonylureas	Thiazolidinediones	Sulfonylureas	Thiazolidinediones
15-19	7	1	NA	1
20-24	10	2	1	2
25-29	26	1	NA	1
30-34	53	3	3	3
35-39	97	9	6	8
40-44	154	22	13	21
45-49	236	24	24	22
50-54	255	32	25	31
55-59	232	28	33	24
60-64	212	32	25	27
65-69	176	24	23	21
70-74	171	14	11	12
75-79	72	6	9	5
80-84	24	2	4	2
85-89	8	NA	2	NA
90-94	1	NA	1	NA

Comparison: Sulfonylureas vs Thiazolidinediones - **Outcome**: Eye Disorders

Age group	Unmatched		Matched	
	Sulfonylureas	Thiazolidinediones	Sulfonylureas	Thiazolidinediones
15-19	7	1	NA	1

© 2018 Vashisht R et al. *JAMA Network Open*.

20-24	10	2	NA	2
25-29	26	1	1	1
30-34	53	3	4	3
35-39	97	9	8	8
40-44	154	22	14	21
45-49	236	24	22	22
50-54	255	32	23	30
55-59	232	28	31	24
60-64	212	32	25	28
65-69	176	24	26	20
70-74	171	14	11	12
75-79	72	6	8	5
80-84	24	2	4	2
85-89	8	NA	2	NA
90-94	1	NA	NA	NA
**Comparison**: DPP4 inhibitors vs Thiazolidinediones - **Outcome**: HbA1c				
Age group	Unmatched		Matched	
	DPP4 inhibitors	Thiazolidinediones	DPP4 inhibitors	Thiazolidinediones
15-19	5	1	1	1
20-24	4	2	1	2
25-29	8	1	2	1
30-34	28	5	3	5
35-39	57	15	11	14
40-44	90	22	10	17
45-49	149	29	23	24
50-54	207	38	34	32
55-59	185	35	35	27
60-64	160	37	33	32
65-69	144	25	22	20
70-74	140	18	14	17
75-79	69	11	8	10
80-84	26	3	7	3
85-89	5	NA	1	NA
90-94	3	NA	NA	NA
**Comparison**: DPP4 inhibitors vs Thiazolidinediones - **Outcome**: Myocardial Infarction				
Age group	Unmatched		Matched	
	DPP4 inhibitors	Thiazolidinediones	DPP4 inhibitors	Thiazolidinediones
15-19	5	1	3	1
20-24	4	2	2	2
25-29	8	1	2	1
30-34	28	5	3	5
35-39	57	15	9	13
40-44	90	22	13	19

© 2018 Vashisht R et al. *JAMA Network Open*.

45-49	149	29	19	25
50-54	207	38	33	34
55-59	185	35	36	28
60-64	160	37	35	33
65-69	144	25	24	20
70-74	140	18	15	17
75-79	69	11	8	10
80-84	26	3	7	3
85-89	5	NA	1	NA
90-94	3	NA	1	NA
**Comparison**: DPP4 inhibitors vs Thiazolidinediones - **Outcome**: Kidney Disorders				
Age group	Unmatched		Matched	
	DPP4 inhibitors	Thiazolidinediones	DPP4 inhibitors	Thiazolidinediones
15-19	5	1	1	1
20-24	4	2	1	2
25-29	8	1	2	1
30-34	28	5	3	5
35-39	57	15	11	14
40-44	90	22	10	19
45-49	149	29	21	25
50-54	207	38	31	33
55-59	185	35	39	27
60-64	160	37	36	32
65-69	144	25	26	19
70-74	140	18	13	17
75-79	69	11	7	10
80-84	26	3	5	3
85-89	5	NA	1	NA
90-94	3	NA	1	NA
**Comparison**: DPP4 inhibitors vs Thiazolidinediones - **Outcome**: Eye Disorders				
Age group	Unmatched		Matched	
	DPP4 inhibitors	Thiazolidinediones	DPP4 inhibitors	Thiazolidinediones
15-19	5	1	3	1
20-24	4	2	1	2
25-29	8	1	3	1
30-34	28	5	3	5
35-39	57	15	11	14
40-44	90	22	13	19
45-49	149	29	21	25
50-54	207	38	34	35
55-59	185	35	35	28
60-64	160	37	30	31
65-69	144	25	25	19

© 2018 Vashisht R et al. *JAMA Network Open*.

70-74	140	18	15	17
75-79	69	11	8	10
80-84	26	3	8	3
85-89	5	NA	NA	NA
90-94	3	NA	NA	NA

© 2018 Vashisht R et al. *JAMA Network Open*.

eTable 14. Age Information Before and After Matching for Each Drug Comparison Based on the Data From Stanford University

**Comparison**: Sulfonylureas vs DPP4 inhibitors - **Outcome**: HbA1c				
Age group	Unmatched		Matched	
	Sulfonylureas	DPP4 inhibitors	Sulfonylureas	DPP4 inhibitors
15-19	1	1	NA	NA
20-24	4	1	2	1
25-29	10	2	2	NA
30-34	16	3	4	3
35-39	39	10	4	9
40-44	45	8	10	5
45-49	54	14	5	7
50-54	84	19	10	10
55-59	111	21	14	17
60-64	113	22	10	10
65-69	114	24	18	13
70-74	77	18	7	10
75-79	60	10	5	3
80-84	39	10	2	5
85-89	14	6	3	4
90-94	10	1	2	1
**Comparison**: Sulfonylureas vs DPP4 inhibitors - **Outcome**: Myocardial Infarction				
Age group	Unmatched		Matched	
	Sulfonylureas	DPP4 inhibitors	Sulfonylureas	DPP4 inhibitors
15-19	1	1	NA	1
20-24	4	1	NA	1
25-29	10	2	1	2
30-34	16	3	6	3
35-39	39	10	9	10
40-44	45	8	11	8
45-49	54	14	15	13
50-54	84	19	16	19
55-59	111	21	24	21
60-64	113	22	16	22
65-69	114	24	28	24
70-74	77	18	14	16
75-79	60	10	13	10
80-84	39	10	10	10
85-89	14	6	4	6
90-94	10	1	NA	1
**Comparison**: Sulfonylureas vs DPP4 inhibitors - **Outcome**: Kidney Disorders				
Age group	Unmatched		Matched	
	Sulfonylureas	DPP4 inhibitors	Sulfonylureas	DPP4 inhibitors
15-19	1	1	NA	1
20-24	4	1	1	1

© 2018 Vashisht R et al. *JAMA Network Open*.

25-29	10	2	NA	1
30-34	16	3	3	3
35-39	39	10	10	9
40-44	45	8	9	7
45-49	54	14	9	12
50-54	84	19	18	18
55-59	111	21	26	20
60-64	113	22	13	18
65-69	114	24	19	23
70-74	77	18	19	15
75-79	60	10	9	6
80-84	39	10	8	7
85-89	14	6	3	5
90-94	10	1	NA	1

**Comparison**: Sulfonylureas vs DPP4 inhibitors - **Outcome**: Eye Disorders				
Age group	Unmatched		Matched	
	Sulfonylureas	DPP4 inhibitors	Sulfonylureas	DPP4 inhibitors
15-19	1	1	NA	1
20-24	4	1	2	1
25-29	10	2	3	1
30-34	16	3	4	3
35-39	39	10	11	10
40-44	45	8	14	7
45-49	54	14	10	11
50-54	84	19	12	16
55-59	111	21	20	20
60-64	113	22	11	16
65-69	114	24	20	19
70-74	77	18	15	13
75-79	60	10	8	6
80-84	39	10	3	6
85-89	14	6	1	4
90-94	10	1	1	1

**Comparison**: Sulfonylureas vs Thiazolidinediones - **Outcome**: HbA1c				
Age group	Unmatched		Matched	
	Sulfonylureas	Thiazolidinediones	Sulfonylureas	Thiazolidinediones
15-19	1	NA	NA	NA
20-24	4	NA	NA	NA
25-29	10	1	1	1
30-34	18	NA	2	NA
35-39	37	3	4	3
40-44	43	6	1	4
45-49	57	14	4	9
50-54	88	21	10	12
55-59	107	22	13	12

© 2018 Vashisht R et al. *JAMA Network Open*.

60-64	98	15	11	9
65-69	113	26	14	14
70-74	81	10	4	6
75-79	57	5	5	1
80-84	36	10	4	6
85-89	15	1	1	1
90-94	10	1	4	NA

Comparison: Sulfonylureas vs Thiazolidinediones - **Outcome**: Myocardial Infarction

Age group	Unmatched		Matched	
	Sulfonylureas	Thiazolidinediones	Sulfonylureas	Thiazolidinediones
15-19	1	NA	NA	NA
20-24	4	NA	1	NA
25-29	10	1	NA	1
30-34	18	NA	3	NA
35-39	37	3	7	3
40-44	43	6	3	6
45-49	57	14	9	14
50-54	88	21	17	21
55-59	107	22	18	22
60-64	98	15	19	15
65-69	113	26	24	26
70-74	81	10	11	10
75-79	57	5	7	5
80-84	36	10	8	9
85-89	15	1	3	1
90-94	10	1	4	1

Comparison: Sulfonylureas vs Thiazolidinediones - **Outcome**: Kidney Disorders

Age group	Unmatched		Matched	
	Sulfonylureas	Thiazolidinediones	Sulfonylureas	Thiazolidinediones
15-19	1	NA	1	NA
20-24	4	NA	NA	NA
25-29	10	1	NA	1
30-34	18	NA	1	NA
35-39	37	3	12	3
40-44	43	6	4	5
45-49	57	14	7	13
50-54	88	21	15	21
55-59	107	22	16	21
60-64	98	15	18	14
65-69	113	26	23	23
70-74	81	10	11	9
75-79	57	5	10	4
80-84	36	10	7	10
85-89	15	1	1	1
90-94	10	1	NA	1

© 2018 Vashisht R et al. *JAMA Network Open*.

**Comparison**: Sulfonylureas vs Thiazolidinediones - **Outcome**: Eye Disorders				
Age group	Unmatched		Matched	
	Sulfonylureas	Thiazolidinediones	Sulfonylureas	Thiazolidinediones
15-19	1	NA	NA	NA
20-24	4	NA	NA	NA
25-29	10	1	NA	1
30-34	18	NA	3	NA
35-39	37	3	4	3
40-44	43	6	6	6
45-49	57	14	7	14
50-54	88	21	13	19
55-59	107	22	18	21
60-64	98	15	18	13
65-69	113	26	26	21
70-74	81	10	6	7
75-79	57	5	11	5
80-84	36	10	7	10
85-89	15	1	1	1
90-94	10	1	1	NA

**Comparison**: DPP4 inhibitors vs Thiazolidinediones - **Outcome**: HbA1c				
Age group	Unmatched		Matched	
	DPP4 inhibitors	Thiazolidinediones	DPP4 inhibitors	Thiazolidinediones
15-19	1	NA	NA	NA
20-24	1	NA	1	NA
25-29	2	1	NA	1
30-34	5	NA	4	NA
35-39	10	5	5	5
40-44	10	9	4	4
45-49	22	18	11	10
50-54	33	30	18	19
55-59	26	33	18	13
60-64	31	39	16	21
65-69	36	44	16	16
70-74	37	19	13	13
75-79	14	14	4	8
80-84	10	13	3	6
85-89	7	1	4	1
90-94	1	1	NA	NA

**Comparison**: DPP4 inhibitors vs Thiazolidinediones - **Outcome**: Myocardial Infarction				
Age group	Unmatched		Matched	
	DPP4 inhibitors	Thiazolidinediones	DPP4 inhibitors	Thiazolidinediones
15-19	1	NA	1	NA
20-24	1	NA	1	NA

© 2018 Vashisht R et al. *JAMA Network Open*.

25-29	2	1	2	1
30-34	5	NA	4	NA
35-39	10	5	6	5
40-44	10	9	8	7
45-49	22	18	17	10
50-54	33	30	27	27
55-59	26	33	18	24
60-64	31	39	24	30
65-69	36	44	28	32
70-74	37	19	18	17
75-79	14	14	11	12
80-84	10	13	4	9
85-89	7	1	5	NA
90-94	1	1	1	1

Comparison: DPP4 inhibitors vs Thiazolidinediones - **Outcome**: Kidney Disorders

Age group	Unmatched		Matched	
	DPP4 inhibitors	Thiazolidinediones	DPP4 inhibitors	Thiazolidinediones
15-19	1	NA	1	NA
20-24	1	NA	1	NA
25-29	2	1	1	1
30-34	5	NA	5	NA
35-39	10	5	6	5
40-44	10	9	8	5
45-49	22	18	16	10
50-54	33	30	27	26
55-59	26	33	19	24
60-64	31	39	20	28
65-69	36	44	28	28
70-74	37	19	19	17
75-79	14	14	6	11
80-84	10	13	3	9
85-89	7	1	4	NA
90-94	1	1	1	1

Comparison: DPP4 inhibitors vs Thiazolidinediones - **Outcome**: Eye Disorders

Age group	Unmatched		Matched	
	DPP4 inhibitors	Thiazolidinediones	DPP4 inhibitors	Thiazolidinediones
15-19	1	NA	1	NA
20-24	1	NA	1	NA
25-29	2	1	1	1
30-34	5	NA	4	NA
35-39	10	5	8	5
40-44	10	9	9	6
45-49	22	18	12	11

© 2018 Vashisht R et al. *JAMA Network Open*.

50-54	33	30	24	23
55-59	26	33	18	22
60-64	31	39	16	25
65-69	36	44	23	23
70-74	37	19	14	13
75-79	14	14	6	8
80-84	10	13	4	8
85-89	7	1	4	NA
90-94	1	1	NA	NA

© 2018 Vashisht R et al. *JAMA Network Open*.

eTable 15. Mean, Median and Standard Deviation of HbA_{1c} Values for the Comparison of Sulfonylureas vs DPP4 Inhibitors Across Eight Study Sites

**Truven MarketScan CCAE**				
**Drug**	Before Index date		After Index date	
	Unmatched	Matched	Unmatched	Matched
	AVG (SD)	AVG (SD)	AVG (SD)	AVG (SD)
Sulfonylureas	9.00 (1.85)	9.01 (1.73)	7.89 (1.87)	7.70 (1.92)
DPP4 inhibitors	8.67 (1.70)	8.92 (1.68)	7.58 (1.99)	7.61 (1.98)
**Columbia University**				
**Drug**	Before Index date		After Index date	
	Unmatched	Matched	Unmatched	Matched
	AVG (SD)	AVG (SD)	AVG (SD)	AVG (SD)
Sulfonylureas	10.08 (2.33)	10.02 (2.38)	8.50 (1.95)	8.02 (1.85)
DPP4 inhibitors	8.52 (1.27)	8.57 (1.39)	7.70 (1.77)	7.70 (1.79)
**IQVIA Disease Analyser France**				
**Drug**	Before Index date		After Index date	
	Unmatched	Matched	Unmatched	Matched
	AVG (SD)	AVG (SD)	AVG (SD)	AVG (SD)
Sulfonylureas	10.19 (39.2)	8.44 (1.41)	7.51 (1.86)	7.87 (5.4)
DPP4 inhibitors	8.52 (2.3)	8.53 (1.28)	7.81 (5.08)	7.50 (1.68)
**Truven MarketScan MDCR**				
**Drug**	Before Index date		After Index date	
	Unmatched	Matched	Unmatched	Matched
	AVG (SD)	AVG (SD)	AVG (SD)	AVG (SD)
Sulfonylureas	8.46 (1.48)	8.40 (1.43)	7.42 (1.47)	7.29 (1.81)
DPP4 inhibitors	8.31 (1.29)	8.45 (1.37)	7.48 (4.43)	7.48 (4.44)
**Mt. Sinai**				
**Drug**	Before Index date		After Index date	
	Unmatched	Matched	Unmatched	Matched
	AVG (SD)	AVG (SD)	AVG (SD)	AVG (SD)
Sulfonylureas	9.20 (1.76)	9.03 (1.61)	7.89 (1.90)	7.71 (1.78)
DPP4 inhibitors	8.66 (1.45)	8.67 (1.35)	7.45 (1.48)	7.45 (1.48)
**Optum Clinformatics Data Mart**				
**Drug**	Before Index date		After Index date	
	Unmatched	Matched	Unmatched	Matched
	AVG (SD)	AVG (SD)	AVG (SD)	AVG (SD)
Sulfonylureas	8.77 (4.88)	8.94 (3.82)	7.89 (5.51)	7.87 (6.18)
DPP4 inhibitors	8.48 (3.25)	8.85 (3.26)	7.93 (5.9)	7.93 (5.84)
**Ajou University, South Korea**				
**Drug**	Before Index date		After Index date	
	Unmatched	Matched	Unmatched	Matched

© 2018 Vashisht R et al. *JAMA Network Open*.

	AVG (SD)	AVG (SD)	AVG (SD)	AVG (SD)
Sulfonylureas	7.63 (1.28)	8.25 (1.55)	7.68 (1.47)	7.56 (1.37)
DPP4 inhibitors	7.89 (1.57)	7.99 (1.37)	7.47 (1.19)	7.45 (1.18)
**Stanford University**				
**Drug**	Before Index date		After Index date	
	Unmatched	Matched	Unmatched	Matched
	AVG (SD)	AVG (SD)	AVG (SD)	AVG (SD)
Sulfonylureas	9.22 (1.71)	8.74 (1.59)	7.95 (1.67)	7.89 (1.52)
DPP4 inhibitors	8.85 (1.52)	8.69 (1.76)	7.50 (1.34)	7.50 (1.33)

© 2018 Vashisht R et al. *JAMA Network Open*.

eTable 16. Mean, Median and Standard Deviation of HbA_{1c} Values for the Comparison of Sulfonylureas vs Thiazolidinediones Across Eight Study Sites

**Truven MarketScan CCAE**				
**Drug**	Before Index date		After Index date	
	Unmatched	Matched	Unmatched	Matched
	AVG (SD)	AVG (SD)	AVG (SD)	AVG (SD)
Sulfonylureas	8.96 (1.84)	9.09 (1.73)	7.85 (1.91)	7.82 (1.86)
Thiazolidinediones	8.97 (1.74)	9.13 (1.73)	7.76 (1.82)	7.76 (1.83)
**Columbia University**				
**Drug**	Before Index date		After Index date	
	Unmatched	Matched	Unmatched	Matched
	AVG (SD)	AVG (SD)	AVG (SD)	AVG (SD)
Sulfonylureas	10.00 (2.3)	10.02 (2.51)	8.45 (1.92)	8.50 (1.96)
Thiazolidinediones	9.94 (2.37)	9.23 (1.62)	8.36 (2.05)	8.36 (2.05)
**IQVIA Disease Analyser France**				
**Drug**	Before Index date		After Index date	
	Unmatched	Matched	Unmatched	Matched
	AVG (SD)	AVG (SD)	AVG (SD)	AVG (SD)
Sulfonylureas	8.82 (3.14)	8.65 (1.44)	7.71 (11.49)	7.62 (2.33)
Thiazolidinediones	10.19 (38.97)	8.77 (1.28)	7.52 (2.6)	7.77 (13.31)
**Truven MarketScan MDCR**				
**Drug**	Before Index date		After Index date	
	Unmatched	Matched	Unmatched	Matched
	AVG (SD)	AVG (SD)	AVG (SD)	AVG (SD)
Sulfonylureas	8.48 (1.47)	8.61 (1.66)	7.40 (1.48)	7.33 (1.32)
Thiazolidinediones	8.54 (1.29)	8.55 (1.3)	7.53 (3.81)	7.54 (3.84)
**Mt. Sinai**				
**Drug**	Before Index date		After Index date	
	Unmatched	Matched	Unmatched	Matched
	AVG (SD)	AVG (SD)	AVG (SD)	AVG (SD)
Sulfonylureas	9.10 (1.69)	9.09 (1.68)	7.80 (1.81)	7.80 (1.69)
Thiazolidinediones	8.97 (1.64)	8.90 (1.58)	7.63 (1.82)	7.63 (1.83)
**Optum Clinformatics Data Mart**				
**Drug**	Before Index date		After Index date	
	Unmatched	Matched	Unmatched	Matched
	AVG (SD)	AVG (SD)	AVG (SD)	AVG (SD)
Sulfonylureas	8.83 (17.63)	9.12 (3.22)	7.90 (5.58)	7.84 (4.21)
Thiazolidinediones	8.83 (3.34)	9.09 (4.00)	7.72 (4.00)	7.73 (4.03)
**Ajou University, South Korea**				
**Drug**	Before Index date		After Index date	
	Unmatched	Matched	Unmatched	Matched

© 2018 Vashisht R et al. *JAMA Network Open*.

	AVG (SD)	AVG (SD)	AVG (SD)	AVG (SD)
Sulfonylureas	7.95 (1.63)	8.27 (1.65)	7.71 (1.47)	7.69 (1.41)
Thiazolidinediones	7.97 (1.46)	8.10 (1.27)	7.87 (1.49)	7.79 (1.45)
**Stanford University**				
**Drug**	Before Index date		After Index date	
	Unmatched	Matched	Unmatched	Matched
	AVG (SD)	AVG (SD)	AVG (SD)	AVG (SD)
Sulfonylureas	9.07 (1.67)	9.73 (2.27)	7.96 (1.63)	8.00 (1.49)
Thiazolidinediones	9.03 (2.08)	9.25 (2.29)	7.66 (1.72)	7.66 (1.72)

© 2018 Vashisht R et al. *JAMA Network Open*.

eTable 17. Mean, Median and Standard Deviation of HbA_{1c} Values for the Comparison of DPP4 Inhibitors vs Thiazolidinediones Across Eight Study Sites

**Truven MarketScan CCAE**				
**Drug**	Before Index date		After Index date	
	Unmatched	Matched	Unmatched	Matched
	AVG (SD)	AVG (SD)	AVG (SD)	AVG (SD)
DPP4 inhibitors	8.71 (1.7)	9.03 (1.69)	7.64 (2.05)	7.72 (2.01)
Thiazolidinediones	9.04 (1.75)	9.18 (1.76)	7.86 (1.83)	7.73 (1.91)
**Columbia University**				
**Drug**	Before Index date		After Index date	
	Unmatched	Matched	Unmatched	Matched
	AVG (SD)	AVG (SD)	AVG (SD)	AVG (SD)
DPP4 inhibitors	8.60 (1.36)	8.42 (1.35)	8.02 (1.77)	8.00 (1.77)
Thiazolidinediones	9.94 (2.35)	9.08 (1.65)	8.61 (2.07)	8.55 (2.13)
**IQVIA Disease Analyser France**				
**Drug**	Before Index date		After Index date	
	Unmatched	Matched	Unmatched	Matched
	AVG (SD)	AVG (SD)	AVG (SD)	AVG (SD)
DPP4 inhibitors	8.51 (2.24)	8.61 (1.60)	7.81 (5.24)	7.85 (5.18)
Thiazolidinediones	8.78 (2.9)	8.69 (1.39)	7.77 (11.7)	8.00 (16.00)
**Truven MarketScan MDCR**				
**Drug**	Before Index date		After Index date	
	Unmatched	Matched	Unmatched	Matched
	AVG (SD)	AVG (SD)	AVG (SD)	AVG (SD)
DPP4 inhibitors	8.36 (1.32)	8.57 (1.35)	7.45 (1.53)	7.48 (1.45)
Thiazolidinediones	8.44 (1.25)	8.60 (1.40)	7.51 (1.36)	7.39 (1.37)
**Mt. Sinai**				
**Drug**	Before Index date		After Index date	
	Unmatched	Matched	Unmatched	Matched
	AVG (SD)	AVG (SD)	AVG (SD)	AVG (SD)
DPP4 inhibitors	8.59 (1.33)	8.93 (1.63)	7.60 (1.60)	7.61 (1.69)
Thiazolidinediones	9.22 (1.70)	9.10 (1.66)	8.02 (2.01)	7.82 (1.87)
**Optum Clinformatics Data Mart**				
**Drug**	Before Index date		After Index date	
	Unmatched	Matched	Unmatched	Matched
	AVG (SD)	AVG (SD)	AVG (SD)	AVG (SD)
DPP4 inhibitors	8.65 (19.34)	9.06 (3.45)	7.96 (5.59)	7.98 (4.76)
Thiazolidinediones	8.91 (3.56)	9.16 (4.88)	7.77 (3.65)	7.81 (4.40)
**Ajou University, South Korea**				
**Drug**	Before Index date		After Index date	
	Unmatched	Matched	Unmatched	Matched

© 2018 Vashisht R et al. *JAMA Network Open*.

	AVG (SD)	AVG (SD)	AVG (SD)	AVG (SD)
DPP4 inhibitors	7.69 (1.33)	7.93 (1.33)	7.56 (1.30)	7.59 (1.27)
Thiazolidinediones	8.07 (1.57)	8.24 (1.47)	7.86 (1.49)	7.83 (1.46)
**Stanford University**				
**Drug**	Before Index date		After Index date	
	Unmatched	Matched	Unmatched	Matched
	AVG (SD)	AVG (SD)	AVG (SD)	AVG (SD)
DPP4 inhibitors	8.69 (1.40)	8.98 (1.80)	7.75 (1.31)	7.73 (1.29)
Thiazolidinediones	8.91 (1.67)	9.05 (1.74)	7.85 (1.73)	7.90 (1.73)

© 2018 Vashisht R et al. *JAMA Network Open*.

eTable 18. Number of Patients, Hazard Ratio, Confidence Intervals (CI), *P* Values and Calibrated *P* Values for Each Drug Comparison and Each Outcome Based on Analysis Across All Eight Study Sites.

**Comparison**: Sulfonylureas vs DPP4 inhibitors - **Outcome**: HbA1c						
**Study Site**	**N**	**Hazard Ratio**	**95% CI Lower bound**	**95% CI Upper bound**	**P-Value**	**Calibrated P-Value**
**Truven Marketscan CCAE**	10011	1.04	0.98	1.09	0.20	0.84
**Columbia University**	205	0.62	0.41	0.91	0.02	0.01
**IQVIA Disease Analyser France**	774	0.71	0.58	0.86	0.00	0.00
**Truven Marketscan MDCR**	1661	1.24	1.09	1.40	0.00	0.04
**Mt Sinai**	880	0.87	0.73	1.04	0.12	0.01
**Optum Clinformatics Data Mart**	24777	1.11	1.08	1.15	0.00	0.81
**Ajou University**	576	1.38	0.95	2.02	0.09	0.00
**Stanford University**	98	0.93	0.55	1.57	0.79	0.52
**Comparison**: Sulfonylureas vs DPP4 inhibitors - **Outcome**: Myocardial Infarction						
**Study Site**	**N**	**Hazard Ratio**	**95% CI Lower bound**	**95% CI Upper bound**	**P-Value**	**Calibrated P-Value**
**Truven Marketscan CCAE**	12839	1.17	0.91	1.52	0.22	0.37
**Columbia University**	304	2.14	0.90	5.61	0.10	0.18
**IQVIA Disease Analyser France**	1081	1.00	0.19	5.40	1.00	0.82
**Truven Marketscan MDCR**	2008	1.40	0.95	2.08	0.10	0.19
**Mt Sinai**	1041	0.65	0.35	1.20	0.18	0.08
**Optum Clinformatics Data Mart**	34303	1.10	0.99	1.23	0.09	0.90
**Ajou University**	599	3.00	0.38	60.62	0.39	0.05
**Stanford University**	167	1.00	0.12	8.33	1.00	0.81
**Comparison**: Sulfonylureas vs DPP4 inhibitors - **Outcome**: Kidney Disorders						

© 2018 Vashisht R et al. *JAMA Network Open*.

Study Site	N	Hazard Ratio	95% CI Lower bound	95% CI Upper bound	P-Value	Calibrated P-Value
**Truven Marketscan CCAE**	11558	1.08	0.99	1.17	0.09	0.56
**Columbia University**	281	1.25	0.79	2.00	0.35	0.60
**IQVIA Disease Analyser France**	1056	0.75	0.40	1.38	0.36	0.13
**Truven Marketscan MDCR**	1554	1.04	0.89	1.21	0.66	0.73
**Mt Sinai**	1013	0.76	0.55	1.03	0.07	0.01
**Optum Clinformatics Data Mart**	29060	1.12	1.07	1.17	0.00	0.78
**Ajou University**	594	2.67	1.10	7.43	0.04	0.00
**Stanford University**	147	2.25	1.17	4.61	0.02	0.00
**Comparison**: Sulfonylureas vs DPP4 inhibitors - **Outcome**: Eye Disorders						
**Study Site**	**N**	**Hazard Ratio**	**95% CI Lower bound**	**95% CI Upper bound**	**P-Value**	**Calibrated P-Value**
**Truven Marketscan CCAE**	10925	1.11	1.03	1.20	0.01	0.26
**Columbia University**	269	1.31	0.64	2.75	0.47	0.67
**IQVIA Disease Analyser France**	1083	3.00	0.69	20.47	0.20	0.30
**Truven Marketscan MDCR**	1227	1.10	0.94	1.29	0.24	0.72
**Mt Sinai**	1006	1.18	0.77	1.81	0.45	0.78
**Optum Clinformatics Data Mart**	26460	1.16	1.11	1.21	0.00	0.46
**Ajou University**	599	3.50	1.26	12.35	0.03	0.00
**Stanford University**	135	1.75	0.75	4.38	0.21	0.09
**Comparison**: Sulfonylureas vs Thiazolidinediones - **Outcome**: HbA1c						
**Study Site**	**N**	**Hazard Ratio**	**95% CI Lower bound**	**95% CI Upper bound**	**P-Value**	**Calibrated P-Value**

© 2018 Vashisht R et al. *JAMA Network Open*.

**Truven Marketscan CCAE**	9714	1.10	1.04	1.17	0.00	0.49
**Columbia University**	269	1.13	0.80	1.61	0.48	0.66
**IQVIA Disease Analyser France**	499	1.15	0.91	1.45	0.25	0.44
**Truven Marketscan MDCR**	1440	1.25	1.10	1.43	0.00	0.13
**Mt Sinai**	417	0.74	0.55	0.98	0.04	0.34
**Optum Clinformatics Data Mart**	22953	0.99	0.95	1.02	0.51	0.11
**Ajou University**	175	1.00	0.47	2.12	1.00	NA
**Stanford University**	78	0.85	0.48	1.48	0.57	0.62
**Comparison**: Sulfonylureas vs Thiazolidinediones - **Outcome**: Myocardial Infarction						
**Study Site**	**N**	**Hazard Ratio**	**95% CI Lower bound**	**95% CI Upper bound**	**P-Value**	**Calibrated P-Value**
**Truven Marketscan CCAE**	10887	0.96	0.74	1.26	0.79	0.54
**Columbia University**	400	2.14	0.90	5.61	0.10	0.14
**IQVIA Disease Analyser France**	608	1.50	0.43	5.87	0.54	0.57
**Truven Marketscan MDCR**	1578	0.87	0.60	1.25	0.46	0.35
**Mt Sinai**	517	0.73	0.28	1.80	0.50	0.67
**Optum Clinformatics Data Mart**	28549	1.14	1.01	1.29	0.03	0.64
**Ajou University**	181	NA	NA	NA	NA	NA
**Stanford University**	134	0.00	NA	1.61	NA	NA
**Comparison**: Sulfonylureas vs Thiazolidinediones - **Outcome**: Kidney Disorders						
**Study Site**	**N**	**Hazard Ratio**	**95% CI Lower bound**	**95% CI Upper bound**	**P-Value**	**Calibrated P-Value**
**Truven Marketscan CCAE**	9980	0.95	0.87	1.03	0.22	0.13
**Columbia University**	372	0.96	0.64	1.43	0.84	0.65
**IQVIA Disease Analyser France**	594	1.43	0.73	2.89	0.31	0.36
**Truven Marketscan MDCR**	1272	0.94	0.80	1.10	0.45	0.34

© 2018 Vashisht R et al. *JAMA Network Open*.

**Mt Sinai**	501	0.85	0.56	1.30	0.46	0.73
**Optum Clinformatics Data Mart**	25775	1.09	1.04	1.15	0.00	0.89
**Ajou University**	180	2.50	0.54	17.45	0.30	NA
**Stanford University**	126	2.00	0.99	4.28	0.06	0.04
**Comparison**: Sulfonylureas vs Thiazolidinediones - **Outcome**: Eye Disorders						
**Study Site**	**N**	**Hazard Ratio**	**95% CI Lower bound**	**95% CI Upper bound**	**P-Value**	**Calibrated P-Value**
**Truven Marketscan CCAE**	9636	0.98	0.90	1.06	0.55	0.26
**Columbia University**	326	1.19	0.74	1.91	0.48	0.64
**IQVIA Disease Analyser France**	604	1.33	0.29	6.77	0.72	0.74
**Truven Marketscan MDCR**	1063	1.10	0.94	1.28	0.24	0.73
**Mt Sinai**	472	1.36	0.79	2.39	0.27	0.19
**Optum Clinformatics Data Mart**	23773	1.07	1.02	1.11	0.01	0.66
**Ajou University**	179	3.50	0.85	23.49	0.14	NA
**Stanford University**	121	1.08	0.50	2.32	0.85	0.53
**Comparison**: DPP4 inhibitors vs Thiazolidinediones - **Outcome**: HbA1c						
**Study Site**	**N**	**Hazard Ratio**	**95% CI Lower bound**	**95% CI Upper bound**	**P-Value**	**Calibrated P-Value**
**Truven Marketscan CCAE**	6871	1.05	0.98	1.13	0.14	0.87
**Columbia University**	175	1.11	0.66	1.88	0.69	0.35
**IQVIA Disease Analyser France**	389	1.76	1.34	2.33	0.00	0.00
**Truven Marketscan MDCR**	942	1.12	0.94	1.35	0.20	0.60
**Mt Sinai**	475	1.00	0.76	1.32	1.00	0.58
**Optum Clinformatics Data Mart**	16763	0.97	0.93	1.01	0.17	0.81
**Ajou University**	205	0.40	0.14	0.98	0.06	NA
**Stanford University**	117	0.85	0.48	1.49	0.57	0.42

© 2018 Vashisht R et al. *JAMA Network Open*.

**Comparison**: DPP4 inhibitors vs Thiazolidinediones - **Outcome**: Myocardial Infarction						
**Study Site**	**N**	**Hazard Ratio**	**95% CI Lower bound**	**95% CI Upper bound**	**P-Value**	**Calibrated P-Value**
**Truven Marketscan CCAE**	8197	1.03	0.74	1.44	0.87	0.90
**Columbia University**	237	1.00	0.19	5.40	1.00	0.65
**IQVIA Disease Analyser France**	509	0.75	0.15	3.40	0.72	0.85
**Truven Marketscan MDCR**	1072	1.28	0.69	2.40	0.44	0.56
**Mt Sinai**	554	0.88	0.31	2.44	0.80	0.70
**Optum Clinformatics Data Mart**	21505	1.11	0.96	1.30	0.17	0.36
**Ajou University**	211	NA	NA	NA	NA	NA
**Stanford University**	175	NA	NA	NA	NA	NA
**Comparison**: DPP4 inhibitors vs Thiazolidinediones - **Outcome**: Kidney Disorders						
**Study Site**	**N**	**Hazard Ratio**	**95% CI Lower bound**	**95% CI Upper bound**	**P-Value**	**Calibrated P-Value**
**Truven Marketscan CCAE**	7557	1.04	0.93	1.17	0.45	0.87
**Columbia University**	221	1.14	0.64	2.07	0.66	0.43
**IQVIA Disease Analyser France**	499	1.50	0.62	3.83	0.38	0.27
**Truven Marketscan MDCR**	888	1.04	0.84	1.29	0.70	0.82
**Mt Sinai**	538	1.50	0.93	2.44	0.10	0.26
**Optum Clinformatics Data Mart**	18927	1.01	0.95	1.07	0.87	0.87
**Ajou University**	208	0.50	0.07	2.56	0.45	NA
**Stanford University**	165	0.64	0.23	1.62	0.36	0.29
**Comparison**: DPP4 inhibitors vs Thiazolidinediones - **Outcome**: Eye Disorders						
**Study Site**	**N**	**Hazard Ratio**	**95% CI Lower bound**	**95% CI Upper bound**	**P-Value**	**Calibrated P-Value**

© 2018 Vashisht R et al. *JAMA Network Open*.

**Truven Marketscan CCAE**	7220	1.03	0.93	1.14	0.59	0.80
**Columbia University**	204	0.67	0.22	1.85	0.45	0.16
**IQVIA Disease Analyser France**	508	1.50	0.25	11.39	0.68	0.59
**Truven Marketscan MDCR**	732	0.95	0.76	1.18	0.62	0.46
**Mt Sinai**	529	1.23	0.59	2.60	0.58	0.74
**Optum Clinformatics Data Mart**	17470	0.95	0.90	1.00	0.06	0.67
**Ajou University**	210	1.00	0.19	5.40	1.00	NA
**Stanford University**	145	0.38	0.08	1.30	0.16	0.14

© 2018 Vashisht R et al. *JAMA Network Open*.

eFigure 1. Cohort Construction

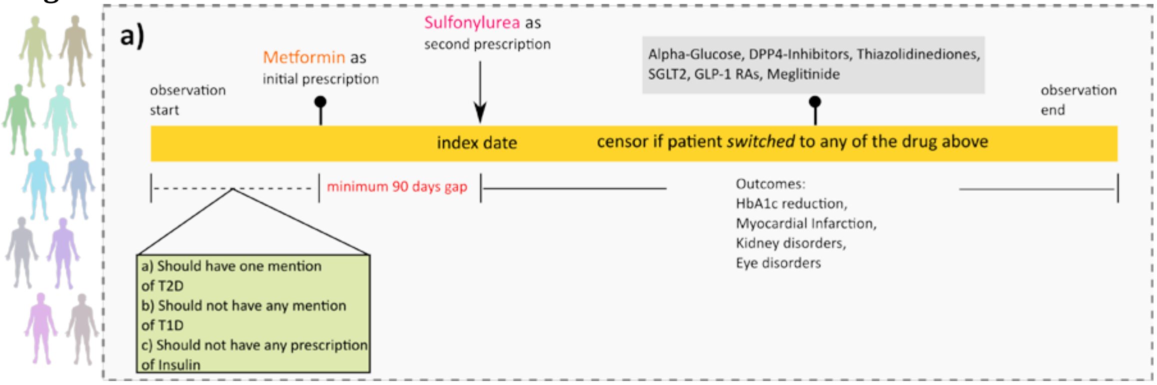

Steps involved in cohort construction and censoring for the time-to-event analysis, using the Sulfonylureas cohort as an example.

A patient is selected based on following conditions.

- Prescription of Second-line drug (Sulfonylureas, DPP4 Inhibitors, or Thiazolidinediones). The date of prescription is considered as the index date relative to which patient is filtered.
- Patient should have a prescription of Metformin at least 90 days prior to index date.
- Patient should have a mention of T2D related codes before index date and no mention of type 1 diabetes codes before the index date. The ICD9-CM or ICD10-CM codes were mapped to their corresponding SNOMED codes to identify patients in the OMOP-CDM schema.
- No prescription of any other T2D related drug among alpha-glucose inhibitors, DPP4 inhibitors, thiazolidinediones, SGLT2 inhibitors, glucagones and meglitinide before the index date including Insulin.
- Reported HbA_{1c} value after the prescription of sulfonylurea. Can have reported incidence of myocardial infarction, kidney disorders and eye disorders.

Patient is right censored depending on the outcome of interest under investigation. The patient is right censored if prescription of any drug among alpha-glucose inhibitors, DPP4 inhibitors, thiazolidinediones, SGLT2 inhibitors, glucagon-like peptide receptor agonists, or meglitinide is observed after the prescription of sulfonylurea. In case of absence of event the complete length of patient's medical record is used as the observational window.

eFigure 1 represents the steps involved in the cohort construction for Sulfonylureas cohort, as an example.

© 2018 Vashisht R et al. *JAMA Network Open*.

eFigure 2. Comparative Effectiveness of Sulfonylureas vs Thiazolidinediones

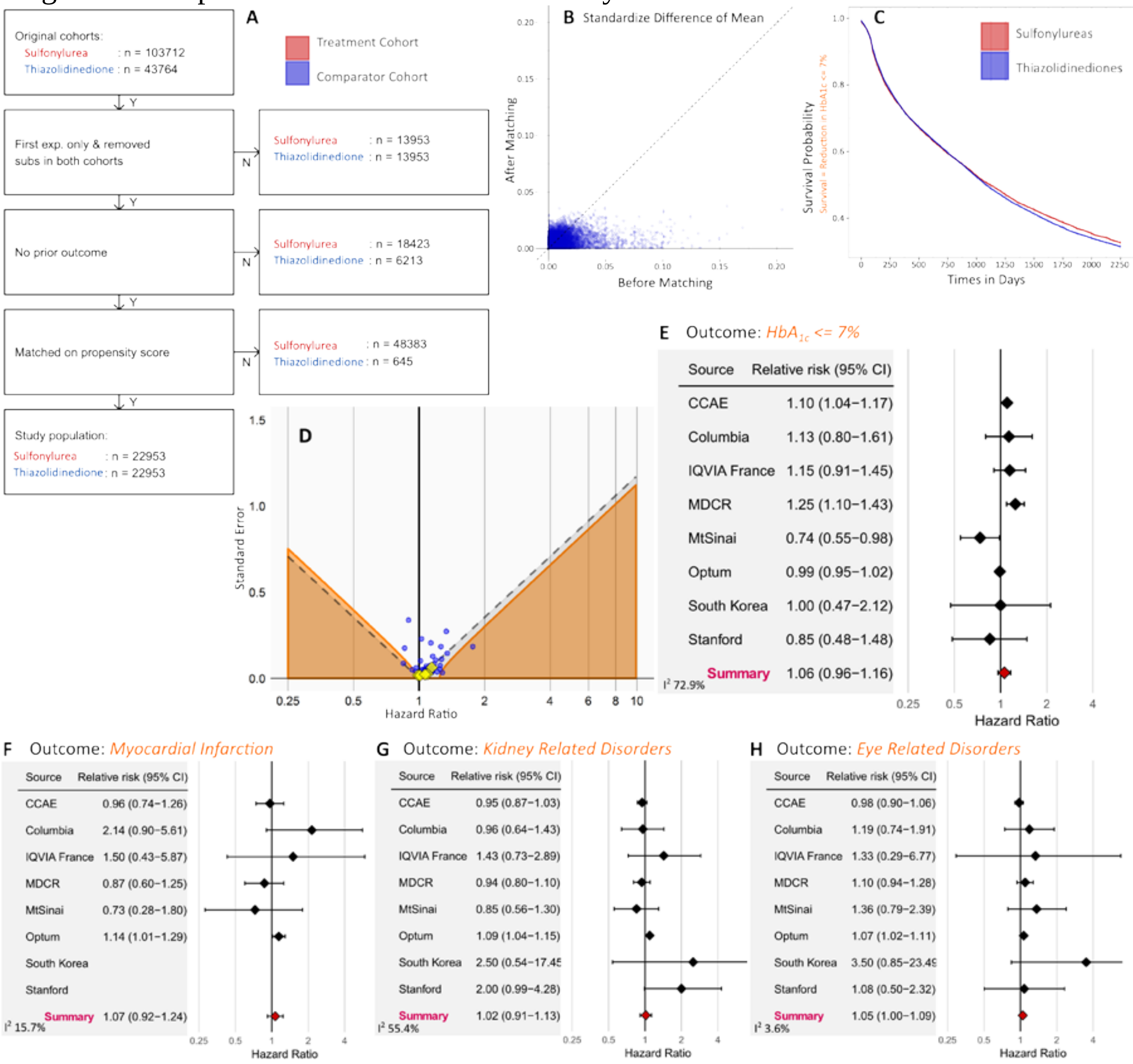

A) Flowchart of matched cohort construction based on the data from Optum, B) covariate balance as standardized mean difference before and after matching based on the data from Optum C) Kaplan-Meier curves for the reduction of HbA_{1c} <= 7% based on the data from Optum D) Empirical calibration plots based on the data from Optum where estimates below the dashed line in gray area have $p < 0.05$ using traditional p-value calculation. Estimates in the orange area have $p < 0.05$ using calibrated p-value calculation. Yellow dots represents outcome and blue dots represent negative controls E) Hazard ratio (HR) of observing a reduction in HbA_{1c} <= 7% after treatment with either sulfonylurea or thiazolidinediones across multiple healthcare systems and consensus effect based on meta-analysis. An HR > 1 mean sulfonylureas are associated with higher hazaed of reducing HbA_{1c} compared to thiazolidinediones. Hazard ratio of observing an event related to F) myocardial infarction G) kidney disorder and H) eye disorder across multiple healthcare systems along with meta-analysis. An HR < 1 means sulfonylureas are associated with a lower hazard of the outcome related to myocardial infarction, kidney and eye disorders. I^2 values for each meta-analysis in E-H are shown in the bottom left of each outcome box.

© 2018 Vashisht R et al. *JAMA Network Open*.

eFigure 3. Comparative Effectiveness of DPP4 Inhibitors vs Thiazolidinediones

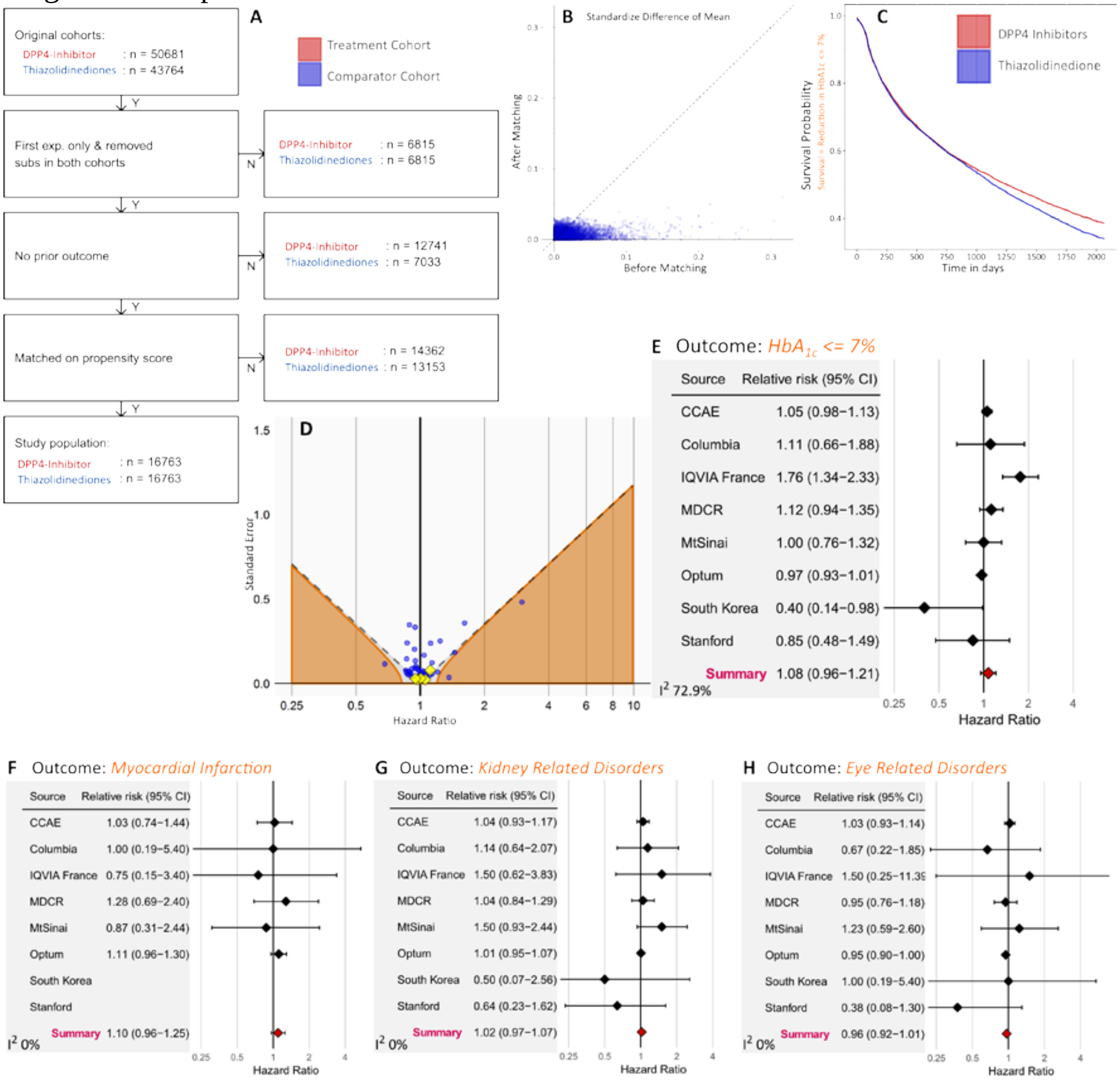

A) Flowchart of matched cohort construction from the data based on Optum, B) covariate balance as standardized mean difference before and after matching from the data based on Optum C) Kaplan-Meier curves for the reduction of HbA_{1c} <= 7% from the data based on Optum D) Empirical calibration plots from the data based on Optum where estimates below the dashed line in gray area have $p < 0.05$ using traditional p-value calculation. Estimates in the orange area have $p < 0.05$ using calibrated p-value calculation. Yellow dots represents outcome and blue dots represent negative controls E) Hazard ratio (HR) of observing a reduction in HbA_{1c} <= 7% after treatment with either DPP4 inhibitors or thiazolidinediones across multiple healthcare systems and consensus effect based on meta-analysis. An HR > 1 means DPP4 inhibitor are associated with higher hazard of reducing HbA_{1c} compared to thiazolidinediones. Hazard ratio of observing an event related to F) myocardial infarction G) kidney disorder and H) eye disorder across multiple healthcare systems along with meta-analysis. An HR < 1 means DPP4 inhibitors are associated with a lower hazard of the outcome related to myocardial infarction, kidney and eye disorders. I^2 values for each meta-analysis in E-H are shown in the bottom left of each outcome box.

© 2018 Vashisht R et al. *JAMA Network Open*.
